# Altered Behavioral Performance and Live Imaging of Circuit-Specific Neural Deficiencies in a Zebrafish Model for Psychomotor Retardation

**DOI:** 10.1371/journal.pgen.1004615

**Published:** 2014-09-25

**Authors:** David Zada, Adi Tovin, Tali Lerer-Goldshtein, Gad David Vatine, Lior Appelbaum

**Affiliations:** 1 The Mina & Everard Goodman Faculty of Life Sciences, Bar-Ilan University, Ramat-Gan, Israel; 2 The Leslie and Susan Gonda Multidisciplinary Brain Research Center, Bar-Ilan University, Ramat-Gan, Israel; 3 Regenerative Medicine Institute, Cedars-Sinai Medical Center, Los Angeles, California, United States of America; University of Pennsylvania, United States of America

## Abstract

The mechanisms and treatment of psychomotor retardation, which includes motor and cognitive impairment, are indefinite. The Allan-Herndon-Dudley syndrome (AHDS) is an X-linked psychomotor retardation characterized by delayed development, severe intellectual disability, muscle hypotonia, and spastic paraplegia, in combination with disturbed thyroid hormone (TH) parameters. AHDS has been associated with mutations in the monocarboxylate transporter 8 (*mct8*/*slc16a2*) gene, which is a TH transporter. In order to determine the pathophysiological mechanisms of AHDS, MCT8 knockout mice were intensively studied. Although these mice faithfully replicated the abnormal serum TH levels, they failed to exhibit the neurological and behavioral symptoms of AHDS patients. Here, we generated an *mct8* mutant (*mct8*−/−) zebrafish using zinc-finger nuclease (ZFN)-mediated targeted gene editing system. The elimination of MCT8 decreased the expression levels of TH receptors; however, it did not affect the expression of other TH-related genes. Similar to human patients, *mct8*−/− larvae exhibited neurological and behavioral deficiencies. High-throughput behavioral assays demonstrated that *mct8*−/− larvae exhibited reduced locomotor activity, altered response to external light and dark transitions and an increase in sleep time. These deficiencies in behavioral performance were associated with altered expression of myelin-related genes and neuron-specific deficiencies in circuit formation. Time-lapse imaging of single-axon arbors and synapses in live *mct8*−/− larvae revealed a reduction in filopodia dynamics and axon branching in sensory neurons and decreased synaptic density in motor neurons. These phenotypes enable assessment of the therapeutic potential of three TH analogs that can enter the cells in the absence of MCT8. The TH analogs restored the myelin and axon outgrowth deficiencies in *mct8*−/− larvae. These findings suggest a mechanism by which MCT8 regulates neural circuit assembly, ultimately mediating sensory and motor control of behavioral performance. We also propose that the administration of TH analogs early during embryo development can specifically reduce neurological damage in AHDS patients.

## Author Summary

In a wide range of brain disorders, mutations in specific genes cause alterations in the development and function of neural circuits that ultimately affect behavior. A major challenge is to uncover the mechanism and provide treatment which is capable of preventing brain damage. Allan-Herndon-Dudley syndrome (AHDS) is a severe psychomotor retardation characterized by intellectual disabilities, neurological impairment and abnormal thyroid hormone (TH) levels. Mutations in the TH transporter MCT8 are associated with AHDS. Mice that lack the MCT8 protein exhibited impaired TH levels, as is the case in human patients; however, they lack neurological defects. Here, we generated an *mct8* mutant (*mct8*−/−) zebrafish, which exhibited neurological and behavioral deficiencies and mimics pathological conditions of AHDS patients. The zebrafish is a simple transparent vertebrate and its nervous system is conserved with mammals. Time-lapse live imaging of single axons and synapses, and video-tracking of behavior revealed deficiencies in neural circuit assembly, which are associated with disturbed sleep and altered locomotor activity. In addition, since the *mct8*−/− larvae provides a highthroughput platform for testing therapeutic drugs, we showed that TH analogs can recover neurological deficiencies in an animal model for psychomotor retardation.

## Introduction

Circuit formation is a fundamental process in the development and operation of the nervous system. Deficiencies in neurogenesis and synaptic connectivity are thought to lie at the root of genetic mental-retardation syndromes [1], [2]. However, their mechanisms and treatment remain mostly indefinite due to the high complexity of brain networks. The Allan-Herndon-Dudley syndrome (AHDS) is a classic example of such a genetic neurological disorder. In AHDS, mutations in the monocarboxylate transporter 8 (*mct8*/*slc16a2*) gene, located on the X chromosome, produce severe psychomotor retardation in young males. AHDS is characterized by a combination of neurological impairments that include hypotonia, spastic paraplegia, lack of speech, and severe cognitive deficiency [3], [4]. In addition, since MCT8 is a thyroid-hormone (TH) transporter, AHDS patients exhibit endocrine alterations in their TH parameters, with decreased plasma concentration of the prohormone 3,5,3′,5′-triiodo-L-thyronine/thyroxine (T4) and increased concentration of the active form 3,5,3′-triiodo-L-thyronine (T3) [3], [4]. In all vertebrates, TH is an essential regulator of development, neurogenesis, growth, and metabolism [5]. In order to function, TH requires efficient transport across the cell membrane because T3 regulates gene transcription by binding to nuclear TH receptors (TRs) [5]. Accordingly, the underlying mechanism of AHDS is thought to involve a defect in the MCT8-dependent neuronal entry of T3, leading to impaired neurological development. However, as is often the case in other retardation syndromes, the location of the altered neuronal circuits and the nature of these deficiencies remain elusive, and adequate treatment is not available.

In humans and rodents, MCT8 is expressed in many tissues including the thyroid gland, the nervous and vascular systems [6]–[8]. In order to elucidate the pathophysiological mechanisms of AHDS, an MCT8 knockout (KO) mouse model was generated. These KO mice replicate the endocrine and metabolic abnormalities found in human patients [9]–[12]. However, they did not display any neurological or behavioral phenotypes. This can be explained by the pronounced expression of the anion transporting polypeptide 1C1 (OATP1C1), a specific T4 transporter, at the blood-brain barrier (BBB) in mice but not in humans, that can compensate for the loss of MCT8 [7], [13], [14]. Thus, development of an alternative animal model that lack MCT8 and demonstrates AHDS-like neurological phenotypes, is essential.

The zebrafish is a powerful model that combines invertebrate-like genetics with vertebrate brain structures, and its transparency allows the visualization of neural circuit dynamics in live animals [15]–[17]. In addition, the hypothalamic-pituitary-thyroid (HPT) axis is conserved in zebrafish [18], and zebrafish larvae have emerged as an attractive model for therapeutic drug screening [19]. In light of these advantages, we have recently isolated the zebrafish *mct8* gene and promoter and showed that, as in humans, zebrafish *mct8* is expressed primarily in the nervous and blood systems [20]. Importantly, zebrafish MCT8 mediates TH uptake in cell lines [21], and knock-down of MCT8 resulted in neurological abnormalities in zebrafish larvae [20].

In this study, in order to determine the function of MCT8 and the mechanisms of AHDS, we used the zinc-finger nuclease (ZFN)-genome editing system to establish an MCT8 mutant (*mct8*−/−) zebrafish model. Using gene quantification and localization assays, as well as time-lapse imaging of single neuronal circuits and synapses in live animals and video-tracking of behavior, we found altered expression of myelin-related genes, circuit-specific deficiencies in axon branching and synaptic density, and altered locomotor activity and sleep in *mct8*−/− larvae. Strikingly, comparative pharmacological assays demonstrated that TH analogs can restore a portion of the neurological phenotypes. These findings suggest a neurological mechanism and treatment for AHDS and, potentially, other related psychomotor retardation disorders.

## Results

### Establishment of ZFN-mediated mct8 mutant zebrafish

**Figure 1:**
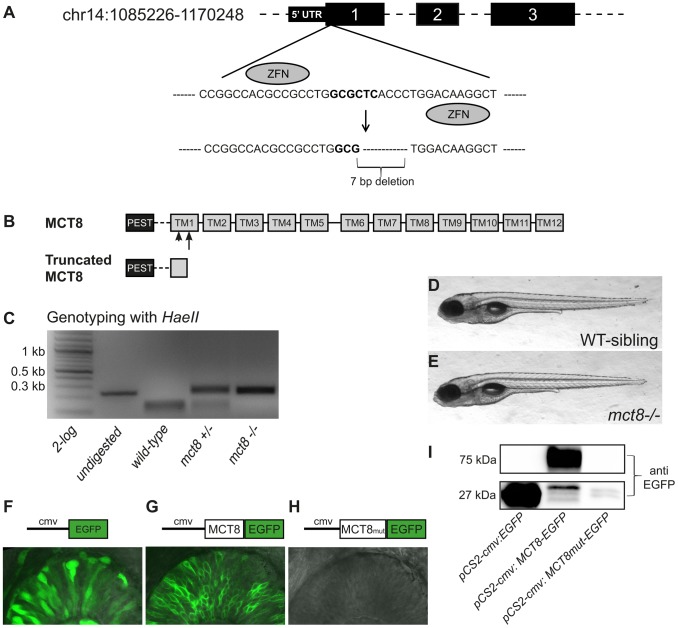
Establishment of ZFN-mediated *mct8* zebrafish mutant. **A**. A pair of ZFNs was designed to target the *HaeII* restriction site (bold) within the first axon of the zebrafish *mct8* gene located on chromosome 14. The targeted mutation resulted in a 7 bp deletion. **B**. The zebrafish MCT8 protein is schematically shown in the upper panel (TM – transmembrane domain). Arrowhead represent the location of the ZFN-mediated deletion and arrow represent the location of the premature stop codon. The truncated MCT8 protein is shown in the lower panel. **C**. Genotyping of WT, *mct8+/−*, and *mct8−/−* embryos. Genomic DNA was amplified, and the 234 bp PCR product (left lane) was digested with *HaeII* restriction enzyme. Complete digestion of WT DNA resulted in two short fragments of 104 bp and 130 bp. An intact 234 bp DNA fragment is shown in *mct8−/−* fish, confirming the introduction of a mutation at the target site. Heterozygous fish exhibit three DNA fragments, indicating both mutated and intact *mct8* alleles. **D, E**. Lateral view of representative 6 dpf WT-sibling and *mct8−/−* larvae. **F–H**. Upper panel: schematic illustration of the *pCS2-cmv:EGFP* (**F**), *pCS2-cmv:MCT8-EGFP* (**G**) and *pCS2-cmv:MCT8mut-EGFP* (**H**) DNA constructs. Lower panel: lateral view of the eye in 30 hpf embryo-injected with *EGFP* (**F**), *MCT8-EGFP* (**G**) and *MCT8mut-EGFP* (**H**) mRNA. **I**. Western blotting using antibody against EGFP. The 27 kDa band represent EGFP and the 72 kDa band represent the fusion protein MCT8-EGFP. Notably, MCT8mut-EGFP protein was not detected.

In order to generate a zebrafish model for AHDS, we targeted a mutation into the genomic *mct8* locus using custom-engineered ZFNs. A pair of ZFNs composed of 5 zinc-finger arrays, which match 15 bp at both sides of the *HaeII* cut site located on the first exon of the gene, were used (Fig. 1A). mRNA coding for each of the two ZFNs was injected into one-cell-stage wild-type (WT) embryos. To verify that the ZFNs system was efficient, on one day post-fertilization (dpf), genomic DNA was extracted from 20 of the injected embryos, and 234 bp genomic fragments that flanked the targeted sequence were amplified. In order to detect the mutation, PCR products were digested using *HaeII* restriction enzyme. An intact DNA fragment was shown in 18 out of the 20 embryos, indicating a mutated allele at the targeted *HaeII* restriction site (Fig. 1C), thus demonstrating the high efficiency of the method. The injected mosaic founder larvae (F0) were raised to adulthood and outcrossed with WT zebrafish. We screened eight F0 fish and found that four transferred the mutation to their F1 offspring. The F1 progeny of selected founder fish were raised to adulthood and 4 out of 6 F1 fish were identified as mutants using tail-clip and genotyping. Of the four F1 heterozygous-mutant (*mct8*+/−) fish, we selected one to establish our mutant zebrafish line. This *mct8*+/− fish harbored a 7 bp deletion mutation that resulted in a frame shift and the incorporation of premature termination codon at amino acid 97, which led to a truncated protein (Fig. 1A, B). The selected F1 fish was outcrossed with WT fish and its progeny were raised to adulthood. The *mct8*+/− F2 adults were intercrossed to produce homozygous *mct8*−/− zebrafish. These *mct8*−/− larvae were viable and fertile and the morphology of the larvae and adult appeared normal (Fig. 1D, E).

In order to confirm that the mutation eliminated MCT8 expression, transient expression studies were performed in larvae. The *mct8* coding sequence was amplified from *mct8−/−* and their WT-siblings. Both mutated and WT coding sequences were fused upstream to EGFP and mRNA of EGFP and the two fusion proteins (*MCT8-EGFP* and *MCT8mut-EGFP*) were injected into one-cell-stage embryos. At 30 hours post fertilization (hpf), somatic and membrane-specific EGFP signal was observed in embryos injected with *EGFP* and *MCT8-EGFP*, respectively. The presence of a membrane pattern confirmed that the zebrafish MCT8 transporter is located in the cell membrane. As predicted, no EGFP expression was found in the *MCT8mut-EGFP* mRNA-injected embryos (Fig. 1F–H). These results were further confirmed using transfection of the three constructs into HEK293T cells followed by western blot. As expected, only in the *pCS2-cmv:MCT8-EGFP* transfected cells, detection with an antibody against EGFP revealed a 75 kDa band that corresponded to the size of the *MCT8-EGFP* fusion protein (Fig. 1I). This 75 kDa band was not detected in *pCS2-cmv:mutMCT8-EGFP* transfected cells. These *in vivo* and *in vitro* results show that the 7 bp deletion in the first exon of *mct8* efficiently eliminates the MCT8 protein.

### The expression of the TH-induced kruppel-like factor 9 and neurogranin genes is not altered in the mct8−/− larvae

**Figure 2:**
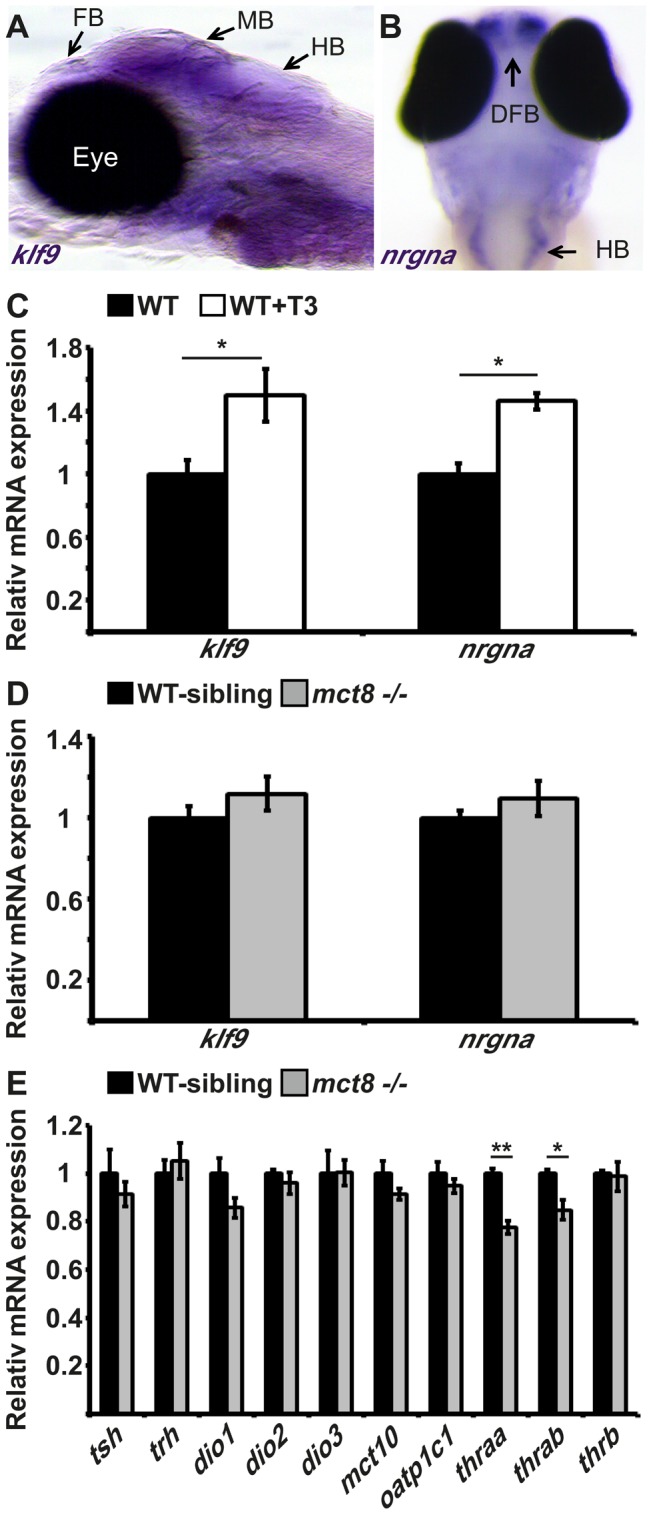
The expression of TH-induced and HPT-axis genes in *mct8*−/− embryos. **A**. The expression pattern of *klf9* in the forebrain (FB), midbrain (MB), and hindbrain (HB) of 6 dpf larvae (lateral view), as detected by whole-mount ISH. **B**. *nrgna* is predominantly expressed in the dorsal forebrain (DFB) and HB in 3 dpf embryo (dorsal view), as detected by whole-mount ISH. **C**. Relative mRNA expression levels of *klf9* and *nrgna* in untreated and T3-treated WT embryos. **D**. Relative mRNA expression of *klf9* and *nrgna* in 3 dpf *mct8−/−* and their WT-sibling embryos. **E**. Relative mRNA expression levels of *tsh*, *trh*, *dio1*, *dio2*, *dio3*, *mct10*, *oatp1c1*, *thraa*, *thrab* and *thrb in* 3 dpf *mct8−/−* and their WT-sibling embryos. Values represented as means±SEM (standard error of the mean). Statistical significance determined by *t*-test: two-sample assuming unequal variances followed by one-Sample Kolmogorov-Smirnov test.

The main mechanism of action of TH is achieved through transcriptional regulation of an array of genes that control neurogenesis, cell growth, and metabolism [5]. Previous studies on mice demonstrated that hyperthyroidism and hypothyroidism in specific regions of the brain alter the expression of specific TH-induced genes, such as *Kruppel-like factor 9* (*klf9*) and *neurogranin* (*RC3/nrgn*) [10], [22]. In order to study the spatial expression pattern and transcript levels of *klf9* and *nrgna* in larvae, whole-mount *in situ* hybridization (ISH) and quantitative real-time PCR (qRT-PCR) assays were performed. Whole-mount ISH experiments showed that while *klf9* is widely expressed in the CNS (Fig. 2A); *nrgna* is specifically expressed in discrete clusters of cells in the dorsal forebrain and hindbrain (Fig. 2B). These patterns of expression are in agreement with previous reports on mammals [10], [23]. To verify that T3 induces *klf9* and *nrgna* expression in zebrafish, T3 (0.5 nM) was administered to embryos beginning at the one-cell stage and until 3 dpf. Transcript levels of *klf9* and *nrgna* were measured using qRT-PCR at 3 dpf. Both *klf9* and *nrgna* expression levels were increased by 49% (*t* = −2.643, *df* = 8, *p*<0.05, Fig. 2C) and 46% (*t* = −5.307, *df* = 8, *p*<0.05, Fig. 2C), respectively, under T3 administration. These results confirm that similar to mammals, *klf9* and *nrgna* are T3-induced genes in zebrafish. To test whether T3 effect inside the cells is attenuated in the absence of a functional MCT8, mRNA levels of *klf9* and *nrnga* were measured in *mct8−/−* and WT-sibling 3 dpf embryos. Although the expression levels of *klf9* and *nrnga* were induced in response to T3 administration (Fig. 2C), their expression levels did not change in *mct8−/−* embryos (Fig. 2D). These results suggest that at 3 dpf, MCT8 does not affect the expression of key TH-induced genes.

### The hypothalamus-pituitary-thyroid axis is not affected by MCT8 elimination

MCT8 facilitates cellular influx and efflux of TH [24], [25], and elimination of this transporter in adult mice reduced TH levels in the brain, as shown by the expression of TH-related genes that are part of the hypothalamus-pituitary-thyroid (HPT) axis [26]. In zebrafish, the knock-down of MCT8, using morpholino-modified antisense oligonucleotides (MO), did not alter the expression of TH-related genes in 2 dpf embryos [20]. To quantify the effect of MCT8 elimination on transcript levels of HPT axis-related genes, we performed qRT-PCR on total mRNA extracted from 3 dpf *mct8−/−* and WT-sibling embryos. In agreement with previous results [20], the mRNA levels of *thyrotropin-releasing hormone* (*trh*), *thyroid-stimulating hormone β* (*tshβ*) and the three deiodinases (*dio1*, *dio2*, *dio3*), did not change in *mct8−/−* compared with their WT siblings (Fig. 2E). These findings raised the possibility that other TH transporters can compensate for the lack of MCT8, and balance TH transport. However, this suggested mechanism is unlikely because the expression patterns of *mct10* and *oatp1c1* generally do not overlap in zebrafish larvae [20]. Nevertheless, we quantified the expression of *mct10* and *oatp1c1* mRNA in 3 dpf *mct8−/−* and WT-sibling embryos. Similar to the expression results of TH-induced and HPT-axis genes, MCT8 elimination did not affect the expression of alternative TH transporters (Fig. 2E). These results suggest that at 3 dpf, right before the endogenous HPT axis becomes functional [18], MCT8 elimination does not affect the expression of HPT axis-related genes. However, these results do not rule out the possibility that TH parameters may be altered in specific tissues and at older developmental stages.

### The expression of thyroid hormone receptor alpha is reduced in mct8−/− embryos

TRs are nuclear ligand-inducible transcription factors that bind T3 and recognize specific DNA sequences, called TH-responsive elements (TREs), in the promoter of TH-induced genes [27]. Three genes encoding TRs are present in zebrafish: *thyroid hormone receptor alpha a (thraa)*, *thyroid hormone receptor alpha b (thrab)* and *thyroid hormone receptor beta* (*thrb*). The two *thra* genes are weakly expressed in embryos and robustly expressed in adult ovaries and testes. At 3 dpf, *thrb* is expressed in the retina, midbrain and hindbrain [27]. To quantify the effect of MCT8 elimination on the transcript levels of TRs genes, qRT-PCR was performed on total mRNA extracted from 3 dpf *mct8−/−* and WT-sibling embryos. We found that mRNA levels of *thraa* and *thrab* were reduced by 23% (*t* = 6.65, *df = 14*, *p<0.001*) and 16% (*t* = 3.4, *df = 12*, *p<0.05*), respectively, in *mct8−/−* compared with their WT siblings (Fig. 2E). In contrast, transcript levels of *thrb* were not changed (Fig. 2E). These results suggest that at 3 dpf, loss of MCT8 does not affect the expression of TH-related genes, including *thrb*. However, it affects the levels of *thraa* and *thrab*. The reduction in expression of these two TH receptors may correlate with reduced TH levels inside the cells.

### The expression of myelin-related genes is altered in mct8−/− developing embryos

**Figure 3:**
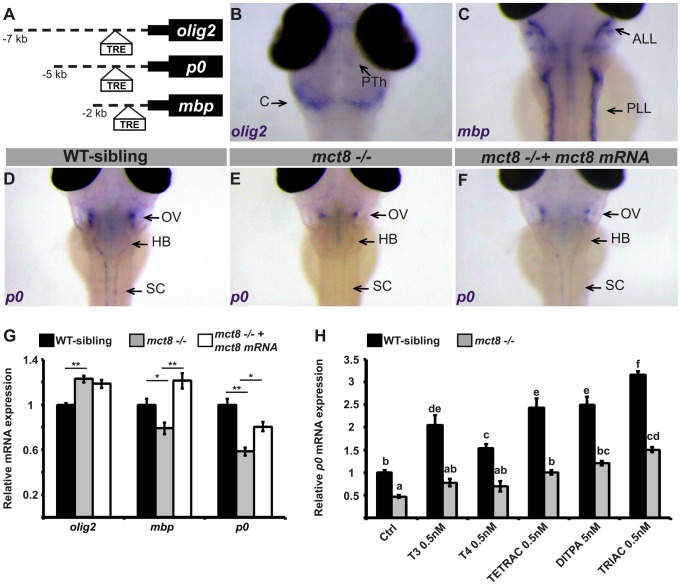
Altered expression of myelin-related genes in *mct8−/−* embryos is recovered by TH analogs. **A**. The location of putative thyroid response elements (TRE) in the 7, 5, and 2 kb promoter regions of the zebrafish *olig2*, *p0*, and *mbp* genes, respectively. **B–F**. Whole-mount ISH experiments detected the spatial mRNA expression in 3 dpf embryos (dorsal views) **B**. *olig2* is predominantly expressed in the prethalamus (PTh) and cerebellum (C). **C**. *mbp* is expressed primarily in the anterior and posterior lateral line (ALL and PLL, respectively). **D**. *p0* is primarily expressed in a cluster of cells above the otic vesicle (OV), the HB, and spinal cord (SC) in WT-sibling embryos. **E**. Low expression levels were detected above the otic vesicle in *mct8−/−* embryos. **F**. The expression pattern of *p0* was recovered in *mct8* mRNA-injected *mct8−/−* embryos. **G**. Relative mRNA expression of *olig2*, *mbp*, and *p0* in 3 dpf WT-sibling, *mct8−/−*, and *mct8* mRNA-injected *mct8−/−* embryos. Values represented as means ±SEM (standard error of the mean). Statistical significance determined by one-way ANOVA followed by a Tukey test (*p<0.05, **p<0.001). **H**. Relative mRNA expression of *p0* in 3 dpf WT-sibling and *mct8−/−* embryos treated with 0.5 nM T3, 0.5 nM T4, 0.5 nM TETRAC, 5 nM DITPA, and 0.5 nM TRIAC compared to control (Ctrl, treated with 5×10^−6^ M NaOH) WT-sibling and *mct8−/−* embryos. Values represented as means ±SEM. Statistical significance determined by two-way ANOVA followed by a Tukey test. Different letters indicate significant difference.

Brain magnetic resonance imaging (MRI) showed markedly delayed myelination and global lack of cerebral white matter in AHDS patients [28]–[30]. The cause of these myelin defects is not clear, and this phenotype was not replicated in MCT8 KO mice. Given the crucial role of TH in oligodendroglial development, maturation, and myelination [31], [32], we hypothesized that a lack of MCT8 alters myelination in the developing zebrafish embryos and that this effect is mediated by TH. Supporting this idea, in mammals, TRs are localized in glial cells expressing *oligodendrocyte lineage transcription factor 2* (*olig2)*, *myelin basic protein* (*mbp*), and *myelin protein zero* (*p0*) [33], [34]. The *olig2* gene is specifically expressed in motor neurons and oligodendrocytes precursor cells in zebrafish and mammals [33], [35]. P0, a major structural protein of myelin [36], [37], and the MBP, which is the most abundant protein component of the myelin sheath [37], [38], are constitutively expressed in mature oligodendrocytes and Schwan cells in the zebrafish CNS and peripheral nervous system (PNS), and are well-established markers for myelination [37]. In mammals, the expression of *mbp* and other myelin-marker genes is induced by TH through binding to TRE present in their regulatory regions [33], [34], [39]. Similarly, in teleost fish, TRE is localized within the promoter of the *mbp* gene [40]. To test whether TRE is present in the promoters of *olig2*, *p0*, and *mbp* also in zebrafish, bioinformatics analysis was performed. In the 7 kb, 5 kb, and 2 kb promoters of *olig2*, *p0*, and *mbp*, respectively, consensus sequences of putative TRE were found approximately 500 bp upstream to the transcription start site (Fig. 3A). These results suggest that these proteins mediate the effect of TH on myelination.

To evaluate the effect of MCT8 elimination on myelination, the spatial distribution of primary and differentiated glial cells was determined by whole-mount ISH. The expression of myelin-related genes is first detected at 2 dpf and the onset of myelination was reported to be at 3 dpf [41]. In 3 dpf embryos, *olig2* was expressed mainly in precursor neural cells in the cerebellum, prethalamus, and spinal cord (Fig. 3B). The *mbp* gene was expressed in mature oligodendrocytes and Schwann cells in the hindbrain, the spinal cord, and in the anterior and posterior lateral line (Fig. 3C). The *p0* was mainly expressed in mature oligodendrocytes in the lateral hindbrain, above the otic vesicle, and in the spinal cord (Fig. 3D). These gene expression patterns confirm previous observations in zebrafish embryos [42]–[44]. Notably, the expression pattern of *p0* was markedly reduced above the otic vesicle and absent from the spinal cord in 3 dpf *mct8−/−* embryos (Fig. 3E). Next, transcript levels of *olig2*, *mbp*, and *p0* were quantified by qRT-PCR in *mct8−/−* and WT-sibling embryos. At 3 dpf, *olig2* mRNA levels were increased by 28% (*F* = 23.12, *df = 2.27*, *p<0.001*), *mbp* mRNA levels were reduced by 27% (*F* = 13.528, *df* = 2.27, *p*<0.05), and *p0* mRNA levels were reduced by 33% (*F* = 22.308, *df* = 2.27, *p*<0.001) in *mct8−/−* compared with their WT siblings (Fig. 3G). These results suggest an increase in the number of precursor glial cells and a decrease in the number of mature glial cells in *mct8−/−* embryos. These findings also suggest deficient myelination in *mct8−/−* embryos. In order to confirm that the observed phenotype is specific to the loss of MCT8, rescue experiments were conducted. At the one-cell stage, *mct8−/−* embryos were injected with *mct8* mRNA. At 3 dpf, the expression levels of *mbp* and *p0* mRNA were increased by 42% (*F* = 13.528, *df* = 2.27, *p*<0.001) and 22% (*F* = 22.308, *df* = 2.27, *p*<0.05), respectively, in *mct8* mRNA-injected *mct8−/−* compared with *mct8−/−* larvae (Fig. 3G). Furthermore, *mct8* mRNA injection did not affect the normal morphology of the embryos but recovered the pattern of *p0* expression (Fig. 3F). These results indicate that *mct8* mRNA can rescue the expression levels of myelin-related genes in *mct8−/−* larvae, suggesting that the myelin phenotype is a specific result of MCT8 deficiency. Furthermore, alterations in the expression of these genes, which control oligodendrocyte differentiation and myelination [41] as well as axon growth and regeneration [45], suggest that deficiencies in myelination and circuit formation are the cause of altered behavioral performance in AHDS patients and could be a suitable target for therapeutic drugs.

### Putative therapeutic drugs: TH analogs can restore the expression of myelin marker in mct8−/− larvae

The therapeutic options for AHDS patients are limited. In order to exploit the zebrafish model, which is ideally suited for testing potential therapeutic molecules, we conducted a comparative pharmacological assay and evaluated the therapeutic potential of three TH analogs: 3,5,3′,5′-tetraiodothyroacetic acid (TETRAC/TA4), 3,3′,5-triiodothyroacetic acid (TRIAC/TA3), and 3,5-diiodothyropropionic acid (DITPA). *In vitro* and *in vivo* studies have demonstrated that these TH analogs can enter the cells independently of the presence of MCT8 [46], [47]. Furthermore, the effect of the T4 analog TETRAC, the T3 analog TRIAC, and the TH receptor agonist DITPA on TH-dependent gene expression and serum TH parameters, has been evaluated in MCT8-KO mice [48]–[50]. However, due to the lack of neurological symptoms, MCT8-KO mice could not be used to monitor the effect of putative drugs on neural recovery.

To monitor the putative therapeutic effect of TH analogs on the mechanism of myelination, *mct8−/−* and WT-sibling embryos were exposed to 0.5 nM T3, T4, TETRAC, TRIAC, and 5 nM DITPA. These component concentrations were chosen based on pre-calibration assays, where the highest dose that did not affect pigmentation and the general morphology of the embryos were selected (see Materials and Methods). Drugs were administered into the egg-water immediately after egg fertilization for three consecutive days. At 3 dpf, mRNA levels of *p0* were quantified by qRT-PCR. This gene was selected because it exhibited the most significant reduction in expression in *mct8*−/− embryos (Fig. 3D–G) and is co-localized with *mct8* (Fig. S1). Similar to our previous results (Fig. 3G), the expression levels of *p0* were reduced by 54% in *mct8−/−* compared with their WT-siblings (*F* = 50.533, *df* = 6.48, *p*<0.001, Fig. 3H). Remarkably, mRNA levels were increased and fully rescued in TETRAC-, DITPA-, and TRIAC-treated *mct8−/−* embryos compared with untreated control *mct8−/−* embryos (Ctrl: 0.46±0.02; TETRAC: 1.01±0.07; DITPA: 1.18±0.1; TRIAC: 1.5±0.07, *p*<0.001; Fig. 3H). In contrast, the expression levels of *p0* in T3- and T4-treated *mct8−/−* embryos were not changed compared with untreated control *mct8−/−* embryos, indicating that TH transport into the cells is altered (Fig. 3H). These results demonstrate that TH analogs can enter into the cells and bypass MCT8. Furthermore, the expression levels of *p0* increased in WT-sibling embryos treated with both TH and TH analogs compared with untreated control WT-sibling embryos (ctrl: 1±0.05; T3: 2.05±0.17; T4: 1.5±0.08; TETRAC: 2.4±0.2; DITPA: 2.5±0.09; TRIAC: 3.15±0.2, *p*<0.001; Fig. 3H). These results were expected because THs are well known inducers of myelin-related processes [31]–[34]. Altogether, these results suggest that loss of MCT8 affects TH transport into the cells and that TH analogs can restore the expression of myelin-related genes. Furthermore, a similar drug mechanism may also be applied in AHDS patients.

### MCT8 mutant exhibits reduced locomotor activity and altered responses to light and dark transitions

**Figure 4:**
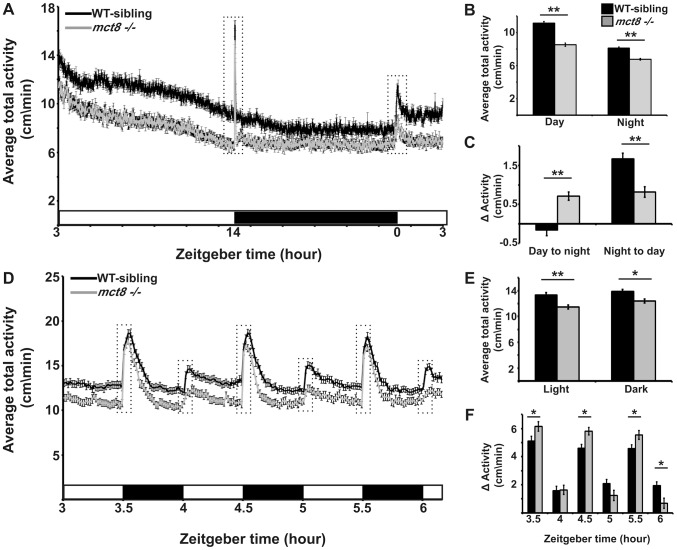
MCT8 mutant exhibits reduced locomotor activity and altered responses to light/dark transitions. Locomotor activity recording was performed in 6 dpf *mct8−/−* larvae and their WT siblings throughout a daily cycle under a 14 h light/10 h dark cycle (**A–C**), or during 3 h of 30 min light/30 min dark intervals (**D–F**). White and black horizontal boxes represent light and dark periods, respectively. Average total activity of each genotype was measured as the average distance movement in 1 min (**A** and **D**). Dotted boxes represent 1 h and 5 min (in **A** and **D**, respectively) before and after the light-to-dark and dark-to-light transitions. The average total activity of each genotype was measured during day and night as well as during short light and dark periods (**B** and **E**, respectively). Differences in the average total activity of each genotype were calculated by comparing 1 h after and 1 h before the day-to-night and night-to-day transitions, as well as by comparing 5 min after and 5 min before light-to-dark and dark-to-light transitions (**C** and **F**, respectively). Values are represented as means±SEM (standard error of the mean). Statistical significance determined by *t*-test: two-sample assuming unequal variances (*p<0.05, ** *p<0.001*).

Deficiency in mobility and voluntary movements is a hallmark of AHDS, and includes the inability to sit, stand, or walk independently, as well as slow psychomotor reaction to sensory input [30]. These symptoms in humans together with the myelin-related phenotype we found in zebrafish, prompted us to test whether behavioral performance was altered in *mct8−/−* larvae. Using a video-tracking behavioral system that can monitor the locomotor activity of dozens of larvae simultaneously [51], [52], the rhythmic activity of *mct8−/−* (n = 144) and WT-sibling larvae (n = 144) was monitored during day and night. Under light/dark conditions (light: 14 h, dark: 10 h), both genotypes exhibited rhythmic activity that peaked during the day. Importantly, during day and night time, the locomotor activity of *mct8*−/− larvae was reduced by 27% (*t* = 8.83, *df* = 286, *p*<0.001) and 21% (*t* = 7.13, *df* = 272, *p*<0.001), respectively, compared to their WT siblings (Fig. 4A, B), indicating reduced overall activity in *mct8*−/− larvae. This reduction of overall activity was partially due to reduced ability to reach maximum velocity in *mct8−/−* larvae. During both day and night the maximum velocity per minute was reduced by 26% (*t* = 8.4, *df* = 286, *p*<0.001) and 24% (*t* = 9.58, *df* = 280, *p*<0.001), respectively, in *mct8−/−* compared to their WT sibling larvae (Fig. S2). Next, we analyzed the day/night transition states. Typically, larvae exhibit a burst of activity, followed by significant change in locomotor activity in response to day-to-night (zeitgeber time, ZT14) and night-to-day (ZT0) transitions. During the first hour following the day-to-night transition (ZT15), WT-sibling larvae reduced their activity and, in contrast, *mct8*−/− larvae increased their activity compared with one hour before the transition (ZT14, *mct8−/−*: 0.7±0.1 cm/min, WT sibling: −0.16±0.15 cm/min; *t* = −4.8, *df* = 268, *p*<0.001; Fig. 4C). Similar comparison was made for the night-to-day transition (ZT1 vs. ZT0). While WT-sibling larvae exhibited the expected elevation in activity following the transition to day, *mct8−/−* larvae maintained relatively low activity levels (*mct8−/−*: 0.8±0.13 cm/min, WT sibling: 1.66±0.15 cm/min; *t* = 4.26, *df* = 285, *p*<0.001; Fig. 4C), indicating differential responses to day/night transitions.

The apparent altered response of *mct8−/−* larvae to day/night transitions could be affected by the circadian time, the light/dark transitions, or both. To test the response of *mct8−/−* larvae to light and dark stimuli, we exposed the larvae to three cycles of alternating 30 min periods of light and darkness during the day (ZT3–ZT6). Both *mct8−/−* (n = 139) and WT siblings (n = 139) responded to light and dark stimuli with robust changes in locomotor activity, as previously shown [51], [53]. Confirming our finding of reduced activity in *mct8−/−* larvae (Fig. 4A, B) during both light and dark periods, *mct8−/−* larvae were 14.2% (*t* = 4.26, *df* = 271, *p*<0.001) and 10.4% (*t* = 3.19, *df* = 272, *p*<0.05) less active compared with their WT siblings (Fig. 4D, E), respectively. Furthermore, analysis of the behavior during the light-to-dark transitions (comparison of the activity 5 min before and after the transition state) showed that the response to dark stimuli was increased in the *mct8*−/− compared with the WT-sibling larvae (*mct8−/−*: 6.15±0.3, 5.8±0.26, 5.54±0.3 cm/min, WT-sibling: 5.1±0.3, 4.6±0.3, 4.6±0.26 cm/min, *p*<0.05; Fig. 4D, F). Notably, this tendency was repeated in all three light/dark cycles. These altered reactions to external stimuli are reversed when comparing the difference in activity between the light and dark phases flanking the dark-to-light transitions. In the third dark-to-light transition (ZT6), the response to light stimuli was decreased in the *mct8*−/− compared with the WT-sibling larvae (*mct8−/−*: 0.7±0.35 cm/min, WT-sibling: 1.93±0.27 cm/min, *p*<0.05; Fig. 4D, F). These behavioral responses to light/dark transitions are in agreement with the behavioral responses found during the day/night transitions. Altogether, these results show reduced baseline locomotor activity and altered behavioral response to light/dark transitions in *mct8*−/− larvae. These findings suggest that MCT8 is involved in the mechanism that regulates locomotor activity and behavioral response to external stimuli. The neurological basis for these deficient behaviors might be conserved between zebrafish and AHDS patients.

### mct8−/− larvae sleep more and their sleep is fragmented

**Figure 5:**
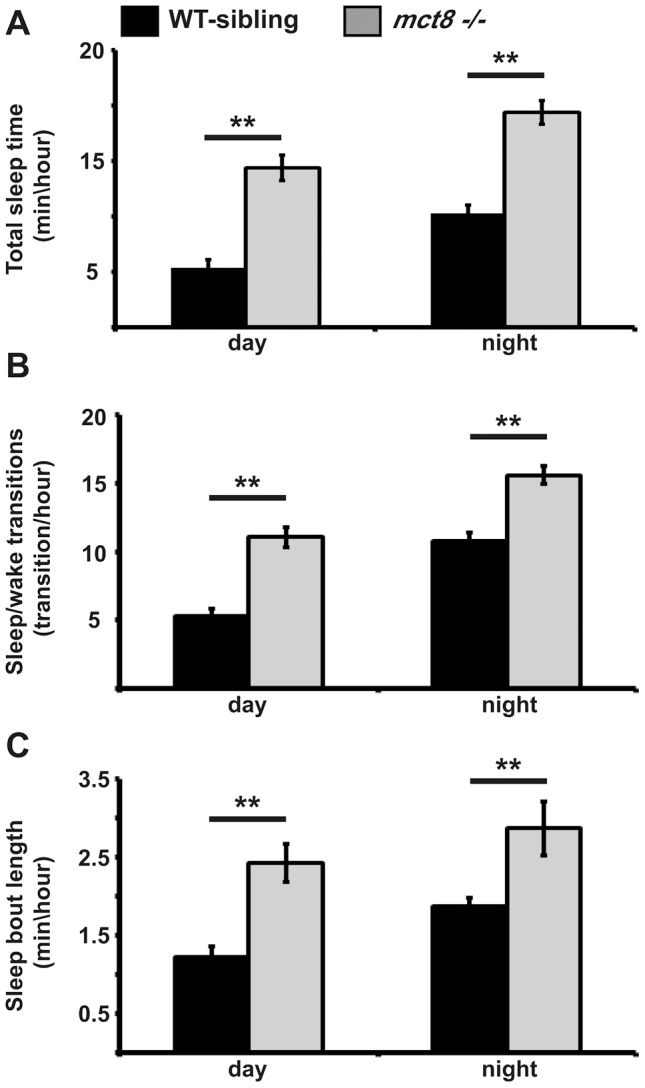
Sleep architecture of *mct8−/−* larvae. **A–C**. Recording of sleep was performed in 6 dpf *mct8−/−* and WT-sibling larvae during 24 h under a 14 h light/10 h dark cycle. Total sleep time (**A**), the number of sleep/wake transitions (**B**) and sleep-bout length (**C**) monitored in *mct8−/−* and WT-sibling larvae. Values are represented as means±SEM (standard error of the mean). Statistical significance was determined by *t*-test: two-sample assuming unequal variances (** *p<0.001*).

The deficiencies in locomotor activity of zebrafish during the night and reports on abnormal sleep in AHDS patients (unpublished results) prompted us to characterize the sleep properties of *mct8*−/− larvae. In the last decade, zebrafish have emerged as an attractive model to study sleep and sleep disorders [54]–[58]. In zebrafish larvae, at least one minute of immobility, which is associated with an increase in arousal threshold, is defined as sleep [51], [55]. To understand the effect of MCT8 elimination on sleep, we monitored sleep architecture in *mct8−/−* larvae (n = 144) and their WT siblings (n = 144) during day and night. As expected, both groups slept more during the night (Fig. 5A). However, sleep time was increased in *mct8−/−* larvae during the 24 h period. Specifically, during day and night, *mct8−/−* larvae slept 2.6- and 1.8-fold more compared with their WT siblings, respectively (*t* = −9.01, *df* = 208, *p*<0.001; *t* = −8.82, *df* = 246, *p*<0.001, Fig. 5A). Furthermore, in order to study sleep consolidation, we monitored the number of transitions between wake and sleep states and sleep-bout length during day and night. In both strains, the number of sleep/wake transitions increased during the night compared with daytime. Of note, the number of transitions was higher by 2 and 1.4 fold during day and night, respectively, in the *mct8−/−* compared with the WT-sibling larvae (*t* = −6.16, *df* = 26, *p*<0.001; *t* = −6.3, *df* = 15, *p*<0.001, Fig. 5B). In addition, the number of transitions during the day in *mct8−/−* larvae was similar to the number of transitions during the night in WT siblings (Fig. 5B), suggesting night-like fragmented sleep in *mct8−/−* larvae during the day.

In order to examine whether increased total sleep time reflects increased ability to maintain long sleep periods, sleep-bout length was analyzed. Sleep-bout length was higher by 2 and 1.5 fold during day and night, respectively, in the *mct8−/−* compared with the WT-sibling larvae (*t* = −6.2, *df* = 24, *p*<0.001; *t* = −8.4, *df* = 12, *p*<0.001, Fig. 5C). These results show that loss of MCT8 increases sleep time and sleep fragmentation during night and day. This is the first evidence of a sleep disorder in MCT8-deficient animals. Taking into account that TSH and TH are rhythmically secreted [59], [60] and their levels are altered in MCT8-KO mice and AHDS patients, sleep patterns should be further investigated in human patients.

### MCT8 elimination does not affect the muscle structure

MCT8 deficiency in human patients affects muscle tone and locomotor activity. The cause for these deficiencies is thought to be neurological [3], [4]. To test whether the reduction in locomotor activity in *mct8*−/− larvae is associated with altered muscle structure, the morphology and development of the fish muscles were studied. At 3 and 6 dpf, the expression pattern of *myoD* and F59, both well-established markers for muscle development [61], [62], was examined in *mct8−/−* and WT-sibling embryos. Confirming our previous finding in MCT8 morphants [20], the pattern of expression of *myoD* and F59 were similar in *mct8*−/− and WT-sibling embryos (Fig. S3). The normal general morphology (Fig. 1D, E) and muscle organization (Fig. S3) observed in *mct8−/−* larvae suggest that altered neuronal circuits are the cause for the deficient behavior.

### Loss of MCT8 reduces synaptic density in axonal arbors of the motor neuron

**Figure 6:**
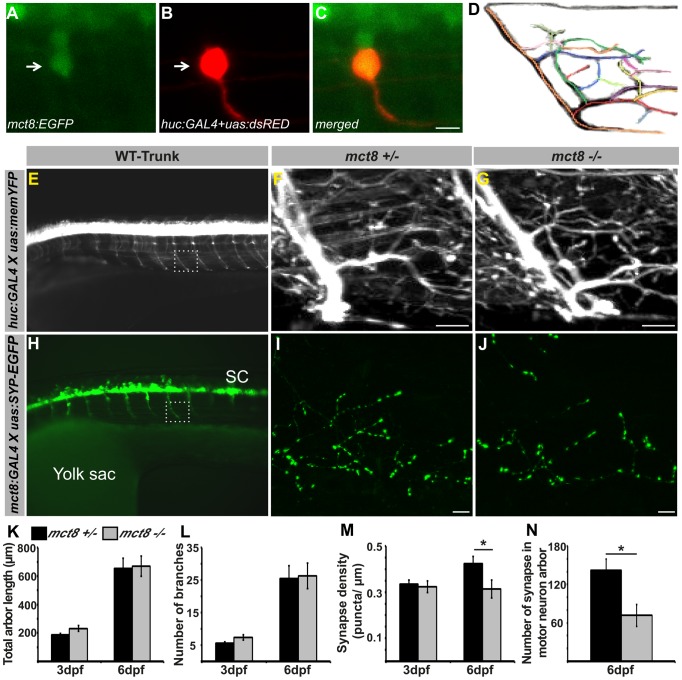
Loss of MCT8 reduces synaptic density in axonal arbors of the motor neuron. **A–C**. Confocal imaging of a 2 dpf live *tg*(*mct8:EGFP*) embryo co-injected with *huc:GAL4* and *uas:tRFP* constructs revealed co-localization of *mct8* (green) and the *huc* pan-neural marker (red) in a motor neuron. **D**. Schematic illustration of an axonal arbor in a motor neuron. Each color represents a single branch that was subjected to ImageJ software analysis. **E**. Lateral view of a 3 dpf *tg(huc:GAL4Xuas:memYFP)* embryo. memYFP expression driven by the *huc* promoter is observed in the spinal cord (SC) and in descending motor neurons. The dashed frame marks a single motor neuron that was selected for further comparative studies. High magnification of the framed area is shown in the trunk of 6 dpf *tg(huc:GAL4Xuas:memYFP)/mct8+/−* and *tg(huc:GAL4Xuas:memYFP)/mct8−/−* representative larvae (**F** and **G**, respectively). **H**. Lateral view of a 30 hpf *tg(mct8:GAL4Xuas:SYP-EGFP)* embryo. SYP-EGFP expression driven by the *mct8* promoter is observed in the spinal cord (SC) and in descending motor neurons. In order to compare the number of synapses in *mct8+/−* and *mct8−/−* larvae, single motor-neuron arbors were selected (dashed frame). High magnification of the dashed frame is shown in 6 dpf *tg(mct8:GAL4)/(uas:SYP-EGFP)/mct8+/−* and *tg(mct8:GAL4)/(uas:SYP-EGFP)/mct8−/−* representative larvae (**I** and **J**, respectively). The total arbor length (**K**) and the number of branches (**L**) were measured in 3 and 6 dpf *mct8+/−* larvae and in 3 and 6 dpf *mct8−/−* larvae. **M**. Synapse density in the axons of the motor-neurons was measured along the last 50 µm of a single branch in 3 and 6 dpf *mct8+/−* larvae and in 3 and 6 dpf *mct8−/−* larvae. **N**. The total number of synapses was measured in the motor-neuron arbor of 6 dpf *mct8+/−* and *mct8−/−* larvae. Scale bar = 30 µm. Values represented as means±SEM (standard error of the mean). Statistical significance determined by *t*-test: Two-sample assuming unequal variances followed by one-sample Kolmogorov-Smirnov test, to assume normal distribution (*p<0.05).

In the absence of adequately functioning MCT8, AHDS patients demonstrate severe cognitive deficiencies, low muscle tone, and dystonia, putatively reflecting the effect of TH deprivation on the CNS [3], [4]. Indeed, PAX8-KO mice, which do not produce endogenous THs, demonstrate severe deficiencies in brain development [48]. However, apparently due to a compensatory mechanism used by other TH transporters, no such brain damage was found in MCT8-KO mice [13], [63] and the neuropathological deficiencies of AHDS patients remain elusive. Taking into account the altered expression of myelin-related genes and deficient locomotor activity in *mct8−/−* zebrafish, we hypothesized that motoric and sensory neurological impairment might be found. To directly assess whether the development and plasticity of motor neurons are affected by loss of MCT8, we sought to image fluorescently labeled motor arbors in the trunk of *mct8−/−* live larvae. The *huc* pan-neural promoter is a well-established, robust tool for marking motor and sensory neurons and endogenous HUC co-localized with MCT8 in zebrafish larvae [20]; therefore, it was used to mark MCT8-expressing motor and sensory neurons [64], [65]. In order to confirm that single *huc* promoter-driven motor neurons express *mct8*, *huc:GAL4* and *uas:tRFP* constructs were co-injected into *tg(mct8:EGFP)* one-cell-stage embryos. Next, single *huc* promoter-driven motor neurons were imaged in 2 dpf embryos. As expected, co-localization of *mct8* and the *huc* pan-neural marker was detected in the motor neurons (Fig. 6A–C). We then tested the effect of MCT8 elimination on axon-arbor processing in single motor neurons. Transgenic *tg(huc:GAL4Xuas:memYFP)/mct8+/−* and *mct8−/−* zebrafish were crossed, and single motor neurons were imaged in live progeny at 3 and 6 dpf (Fig. 6D, E). Image analysis revealed that the total length of the arbor branches (Fig. 6F, G, and K) and the number of branches (Fig. 6F, G, and L) were similar in *mct8−/−* (3 dpf: n = 12, 6 dpf: n = 12) and *mct8+/−* larvae (3 dpf: n = 8, 6 dpf: n = 9). These results show that the structure and length of axon arbor in motor neurons are not affected by MCT8 elimination.

Since alteration in outgrowth and branching of motor neurons was not observed in *mct8−/−* larvae, we tested whether MCT8 is a regulator of structural synaptic dynamics. To visualize synapses on axon arbors in live fish, we labeled the neurons with the presynaptic protein synaptophysin (SYP) fused to enhanced green fluorescent protein (SYP-EGFP). This protein is a well-established synaptic marker in zebrafish [15] and was previously used to demonstrate rhythmic structural synaptic plasticity in the axons of hypocretin/orexin neurons [17]. In order to tag synapses in every circuit of interest, we established a stable transgenic line *tg(uas:SYP-EGFP)* expressing SYP-EGFP under the control of *uas*. In order to examine synapses in motor neurons, *tg(mct8:GAL4)* [20] and *tg(uas:SYP-EGFP)* were crossed and their progeny were imaged. In contrast to the wide expression of EGFP in *tg(mct8:EGFP)* larvae [20], in the double transgenic line EGFP was represented specifically in neurons and synapse structures in the axon fibers of 30 hpf embryos (Fig. 6H). Then *tg(mct8:GAL4)*/*tg(uas:SYP-EGFP)*/*mct8−/−* and *tg(mct8:GAL4)*/*tg(uas:SYP-EGFP)*/*mct8+/−* fish lines were generated. At 3 and 6 dpf, synapses in the axonal arbor of single motor neurons were imaged in both *mct8* genotypes. While at 3 dpf, synaptic density was not changed (*mct8+/−*: n = 34, *mct8−/−*: n = 24, Fig. 6M), a reduction of 27% was found in 6 dpf *tg(mct8:GAL4)*/*tg(uas:SYP-EGFP)*/*mct8−/−* larvae (n = 20) compared with the control *tg(mct8:GAL4)*/*tg(uas:SYP-EGFP)*/*mct8+/−* larvae (n = 30, *t* = 2.309, *df* = 48, *p*<0.05, Fig. 6I, J, and M). In addition, a 50% reduction in the total number of SYP-EGFP puncta in the axonal arbor were found in *tg(mct8:GAL4)*/*tg(uas:SYP-EGFP)*/*mct8−/−* 6 dpf larvae (n = 17) compared with the control *tg(mct8:GAL4)*/*tg(uas:SYP-EGFP)*/*mct8+/−* 6 dpf larvae (n = 31, *t* = 2.639, *df* = 46, *p*<0.05, Fig. 6I, J, and N). These results show that loss of MCT8 decreases total synapse number and synaptic density in the axons of the motor neurons in 6 dpf larvae, while it does not impair axonal outgrowth and branching, suggesting that MCT8 affects structural synaptic changes and plays a crucial role in mediating neural signaling between the nervous system and the muscles.

### MCT8 regulates axon branching in sensory neurons

**Figure 7:**
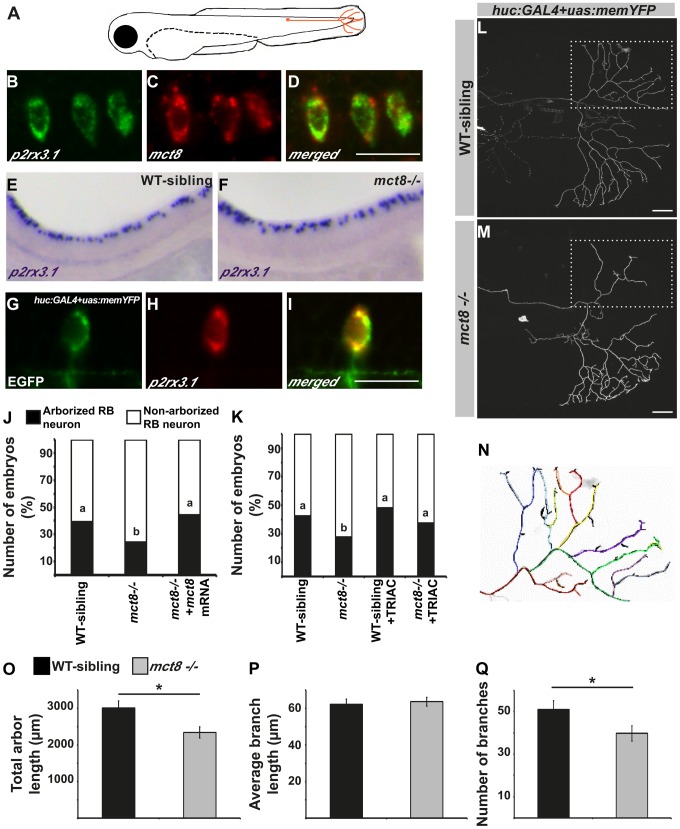
MCT8 regulates axon branching in the Rohon-Beard sensory neurons. **A**. A representative scheme of the Rohon-Beard (RB) sensory neuron location in zebrafish larvae. **B–D**. Double fluorescent ISH in 33 hpf embryos revealed co-localization of *p2rx3.1* (green) and *mct8* (red) in RB cell bodies. **E–F**. Whole mount ISH showed the spatial expression of *p2rx3.1* in the dorsal spinal cord of 2 dpf WT-sibling (**E**) and *mct8−/−* larvae (**F**). **G–I**. Whole-mount ISH and immunofluorescence revealed co-localization of EGFP (green) and *p2rx3.1* (red) in the cell body of an RB neuron in 2 dpf *huc:GAL4+uas:memYFP*-injected embryos. **J**. The percentages of embryos that express *memYFP* in single arborized RB neurons in the tail (black bars), are shown in 2 dpf WT-sibling, *mct8−/−* and *mct8* mRNA-injected *mct8−/−* embryos. Statistical significance was determined by the Chi square test. Different letters indicate significant difference. **K**. The percentages of embryos that express *memYFP* in single arborized RB neurons in the tail (black bars), are shown in 2 dpf WT-sibling, *mct8−/−*, WT-sibling treated with 0.5 nM TRIAC and *mct8−/−* treated with 0.5 nM TRIAC. Statistical significance was determined by the Chi square test. Different letters indicate significant difference. **L, M**. Lateral view of arborized RB-neuron that projects toward the tail in 2 dpf live *mct8−/−* and WT-sibling embryos, which are transiently expressed *huc:GAL4* and *uas:memYFP* constructs. **N**. Schematic illustration of arborized RB sensory neuron. Each color represents a single branch that was subjected to ImageJ software analysis. Filopodia are colored in black. The total length (**O**), average length (**P**), and number of branches (**Q**) measured in *mct8−/−* and WT-sibling embryos. Scale bar = 30 µm. Values represented as means±SEM (standard error of the mean). Statistical significance determined by *t*-test: Two-sample assuming unequal variances followed by one-sample Kolmogorov-Smirnov test, to assume normal distribution (*p<0.05).

The altered expression of myelin-related genes and the disrupted behavioral response to external stimuli in *mct8−/−* larvae suggest that in addition to the motor neurons, sensory neuronal circuits can be altered. In zebrafish larvae, the response to light and touch stimuli and initiation of the first escape response are mediated by the primary Rohon-Beard (RB) sensory neurons. These neurons are located in the dorsal spinal cord and have axons that project toward the hindbrain and the tail [66], [67] (Fig. 7A). In 2 dpf embryos, the RB neurons are fully mature while, at older ages, their axons are gradually abolished and the neurons differentiated to the dorsal root ganglia that have the same sensory functions as the RB neurons [66]. To verify that *mct8* is expressed in RB neurons, double-ISH of *mct8* and *p2rx3.1*, a marker for RB neurons [68], was performed. This staining showed that *mct8* and *p2rx3.1* are co-localized in RB neurons (Fig. 7B–D). In order to test the effect of MCT8 elimination on the pattern and spatial distribution of RB neurons, whole-mount ISH was performed using *p2rx3.1* probe. At 33 hpf, no differences were found between *mct8−/−* and WT-sibling embryos (Fig. 7E and F). However, although the cell bodies of RB neurons were intact, an MCT8-dependent deficiency in axon outgrowth may be present.

In order to explore the specific effects of MCT8 elimination on the dynamics of RB-axon-arbor structure, we imaged single RB neurons in live embryos. The constructs *huc:GAL4* and *uas:memYFP* were co-injected into *mct8−/−* and WT-sibling embryos. This transient expression resulted in mosaic *memYFP* expression in several types of neurons including *p2rx3.1*-positive RB-neurons (Fig. 7G–I). At 2 dpf, *memYFP*-positive embryos were sorted out and among the positive embryos 24% (n = 332) and 39% (n = 303) showed *memYFP* expression in single RB neuron that projected toward the tail in *mct8−/−* and WT-sibling embryos, respectively (*χ2* = 20.337, *df* = 2, *p*<0.001, Fig. 7A, J). These results demonstrate that the number of mature RB neurons that contain axon arbor in the tail is reduced in *mct8−/−* embryos, and suggest that loss of MCT8 altered the development of the axonal arbor of RB neurons. To confirm that the observed differences are specific to MCT8 deficiency, we performed rescue experiments. The constructs *huc:GAL4* and *uas:memYFP* were co-injected with and without *mct8* mRNA into one-cell-stage *mct8−/−* embryos. At 2 dpf, the number of *mct8* mRNA-injected embryos that showed *memYFP* expression in single arborized RB neurons increased to 44% (n = 52, *χ2* = 20.337, *df* = 2, *p*<0.001, Fig. 7J), which is similar to the percentage of embryos observed in the WT-sibling group.

The MCT8-dependent arborization phenotype prompted us to test whether TH analogs can also rescue this neurological deficiency in *mct8*−/− embryos. Two constructs, *huc:GAL4* and *uas:memYFP*, were co-injected into one-cell-stage *mct8−/−* and WT-sibling embryos which were then raised in 0.5 nM TRIAC for two days. TRIAC was selected because it exhibited the most significant effect on the expression of *p0* in WT-sibling and *mct8*−/− embryos (Fig. 3H). Similar to previous observations (Fig. 7J), at 2 dpf, 27.5% (n = 109) and 42.8% (n = 66) of the positive embryos showed *memYFP* expression in single RB neuron that projected toward the tail in *mct8−/−* and WT-sibling embryos, respectively (*χ2* = 4.1, *df* = 3, *p*<0.05, Fig. 7K). Importantly, the number of TRIAC-treated *mct8−/−* embryos that showed *memYFP* expression in single arborized RB neurons increased to 44% (n = 99, *χ2* = 6.77, *df* = 3, *p*<0.05, Fig. 7K). In contrast, no significant differences were found in WT-sibling treated with TRIAC (n = 52). These results show that the TH analog can recover the development of axons in RB neurons of *mct8−/−* embryos.

To elucidate specific axonal deficiencies, we imaged and quantified the total arbor length, the number of branches, and the average branching length of single RB neurons in *mct8−/−* and WT-sibling embryos. Live imaging showed that total arbor length was reduced by 32% in *mct8−/−* (n = 34) compared with WT-sibling embryos (n = 23, *t* = 2.030, *df* = 55, *p*<0.05, Fig. 7L–O). This decrease in arbor length could be the result of a decrease in the number of branches per axon arbor, a decrease in branch length, or by a combination of both processes. Intriguingly, we found that the average length of a single branch was similar in both genotypes (Fig. 7L–N and P); however, the number of branches was reduced by 22% in *mct8−/−* (n = 34) compared with WT-sibling embryos (n = 23, *t* = 2.783, *df* = 55, *p*<0.05, Fig. 7L–N and Q). These results show that MCT8 is essential for the mechanism that controls RB-axon branching.

### Filopodia dynamics in RB-axons is reduced in mct8−/− embryos

**Figure 8:**
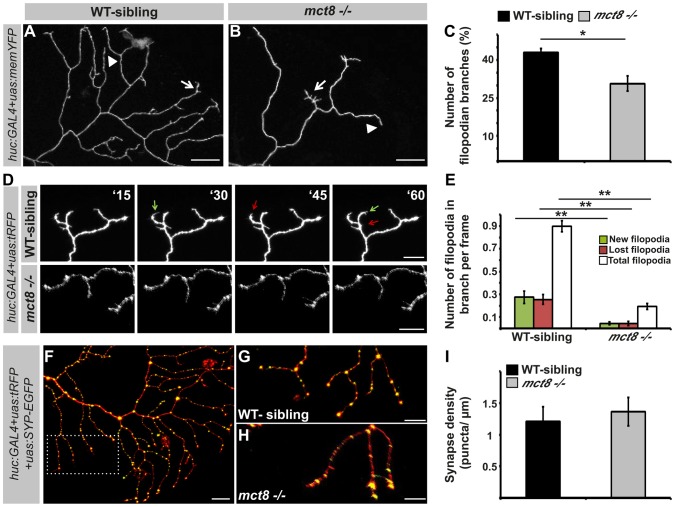
MCT8 reduces filopodia dynamics in the axons of RB neurons. **A–B**. High magnification views of the dotted area shown in Fig. 7L and 7M, respectively. Arrows mark branches that contain filopodia and arrowheads mark branches that lack filopodia. **C**. Number of filopodian branches in *mct8−/−* and WT-sibling embryos. **D**. Time-lapse live imaging of axon arbor of RB sensory neuron (15 min intervals during 135 min). A representative series of images that were taken every 15 min in live *mct8−/−* and WT-sibling embryos is shown. Filopodia dynamics is defined as the number of new (green arrows) and lost (red arrows) filopodia per branch over time. **E**. Filopodia dynamics per branch during 150 min. **F–H**. Live imaging of synapses in the axons of the RB sensory neurons. **F**. Lateral view of axons and synapses marked with tRFP and SYP-EGFP, respectively. The dotted frame marks the area shown in high magnification in **G** and **H**. **I**. Synapse density in the RB-neuron arbor of *mct8−/−* and WT-sibling embryos measured along the last 50 µm of a single branch. Scale bar = 30 µm. Values represented as means ±SEM (standard error of the mean). Statistical significance determined by *t*-test: two-sample assuming unequal variances followed by one-sample Kolmogorov-Smirnov test to assume normal distribution (*p<0.05, ** *p<0.001*).

The growth of RB-axon arbors is a highly dynamic process characterized by the formation of numerous transient and highly dynamic filopodia. Only a small fraction of the filopodia develops into stable branches in the mature arbor [69]. To understand the mechanism which regulates the reduction in the number of branches in the RB-axon arbor of *mct8−/−* embryos, the number of filopodia was quantified. First, to study the effect of T3 on filopodia dynamics in zebrafish larvae, T3 (0.5 nM) was administered to *huc:GAL4/uas:memYFP* injected embryos beginning at the one-cell stage and until 2 dpf. Under T3 administration, the number of filopodian branches per single axon arbor was increased by 25% (WT: n = 25, WT+TRIAC: n = 15, *t* = 2.06, *df* = 24, *p*<0.05, Fig. S4). These results confirm that similar to mammals [70], [71], TH induces filopodia dynamics in zebrafish. Then, the number of filopodian branches was quantified in WT-sibling and *mct8−/−* embryos. At 2 dpf, the number of filopodian branches in axon arbors was reduced by 30% in *mct8−/−* (n = 34) compared with their WT-sibling embryos (n = 23, *t* = 4.188, *df* = 43, *p*<0.05, Fig. 8A–C). In order to track filopodia dynamics in the axon arbors, time-lapse imaging was performed (Video S1). We quantified the number of total, new and lost filopodia per branch. New and lost filopodia were defined when they appeared or disappeared between frames (frame intervals: 15 min), respectively. During 135 minutes, the average number of new and lost filopodia was reduced by 95% and 94% in *mct8−/−* 2 dpf embryos, respectively (both genotypes: n = 10, *t* = 4.151, *df* = 9, *p<0.001* and *t* = 4.453, *df* = 11, *p*<0.001, Fig. 8D, E). In addition, the average number of total filopodia per single branch was reduced by 78% in *mct8−/−* compared with WT-sibling 2 dpf embryos (*t* = 12.716, *df* = 14, *p*<0.001, Fig. 8D, E). These time-lapse imaging experiments show that the dynamic changes of filopodia formation and elimination are reduced in *mct8−/−* embryos. Altogether, these results suggest a mechanism by which reduction in the rate of filopodial turnover leads to a reduced number of stable filopodia that will develop into mature axon branches in *mct8−/−* larvae.

The branching developmental defect might be associated with alteration in synaptic formation because, in other neuronal circuits, synaptogenesis guides the branching of axonal arbors in zebrafish [15]. Therefore, the *huc:GAL4*, *uas:SYP-EGFP*, and *uas:tRFP* constructs were co-injected into *mct8−/−* embryos and their WT siblings. At 2 dpf, synapse density was quantified in the axonal arbor of single RB neurons (Fig. 8F) in both genotypes. Unlike the alteration found in the motor neurons (Fig. 6I–J and M–N), synaptic density in the RB neurons was similar in both genotypes (*mct8−/−*: n = 8, WT-sibling: n = 8, Fig. 8G–I). Altogether, these results show that MCT8 regulates filopodial turnover and axon branching in sensory RB neurons.

## Discussion

Elucidating the pathophysiological mechanisms underlying the inhibition of cognitive and motor activity in patients with psychomotor retardation will improve their therapeutic management. Psychomotor retardation AHDS is an inherited, X-linked, single-gene disorder. In the afflicted population, the *mct8* gene is mutated, and the consequence in humans is altered TH levels and a diverse constellation of psychiatric and neurological symptoms [3], [4], [63]. Based on these symptoms, as well as on research on cell lines and MCT8-KO mice, it was suggested that the loss of MCT8 results in reduced transport of TH into the brain, thus TH signaling is altered and causes deficiencies in CNS development [72]. However, no neurological phenotype was found in MCT8-KO mice, and the mechanism and treatment of the disorder remained enigmatic. In the current research, we established and characterized a ZFN-based zebrafish mutant of MCT8. Transient transgensis and *mct8* mRNA as well as pharmacological rescue experiments have shown that the ZFN-mediated mutation specifically and efficiently altered MCT8 function. Considering the methodological advantages of this transparent vertebrate, the *mct8*−/− zebrafish provides a stable model that allows whole-brain analysis in live animals during all developmental stages. Using genetic manipulations, time-lapse live imaging, and video-tracking of behavior, we found alteration in the expression of myelin-related genes, circuit-specific alteration in circuit formation, and deficient behavior in *mct8*−/− larvae. Comparative pharmacological assays showed that TH analogs can recover a portion of the neurological phenotypes. Thus, the zebrafish provides an attractive model to study the mechanisms and test possible treatments for AHDS specifically and psychomotor retardation in general.

**Figure 9:**
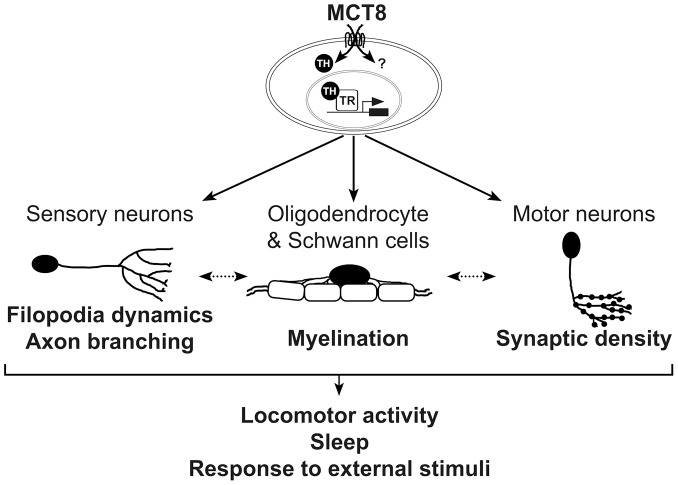
A proposed model for the mechanism underlying MCT8 deficiency. In zebrafish, MCT8 transports TH across the membrane of oligodendrocytes, Schwann cells, and neurons, and regulates gene expression through TRs. Other MCT8-dependent pathways can affect neuronal development, primarily at early embryonic developmental stages (represented by question mark). Loss of MCT8 affects myelination, filopodia dynamics, axon branching, and synaptic density. The deficiencies in axon processes and synapse numbers impaired the number of mature myelinated axons, or alternatively, deficiency in the development of glial cells alters filopodia dynamics, axon branching, and synaptic density (dotted arrow). The combined neural deficiencies alter behavioral performance, including locomotor activity, sleep and response to external stimuli. A portion of these MCT8-dependent deficiencies including the expression of myelin-related gene and axon outgrowth can be recovered by TH analogs. Similar mechanism may be applied in AHDS patients.

The current explanation for the symptoms of AHDS suggests that diminished TH supply during critical stages of brain development alters the expression of the HPT-axis-related and TH-induced genes, and eventually leads to neurological and behavioral abnormalities [63], [73], [74]. Indeed, MCT8-KO mice replicated the abnormal thyroid parameters found in AHDS patients and showed increased serum T3 values and low T4 levels [63], [73], [74]. Furthermore, the level of expression of HPT-axis genes, such as *tsh* and *dio2*, and TH-induced genes, such as *klf9* and *nrgn*, were altered [22], [48]. Intriguingly, these changes in gene expression were not associated with neurological impairments in the MCT8-KO mice [63]. In contrast, the expression levels of *klf9*, *nrgna* and HPT-axis-related genes were not changed in 3 dpf *mct8*−/− zebrafish embryos; nevertheless, apparent neurological and behavioral deficiencies were found. A possible explanation to the lack of gene alteration in *mct8*−/− zebrafish could be the relatively early developmental stage at which the embryos were sampled, when the negative TH feedback loop was still not apparent [18] and endogenous TH production was limited [75]. This unchanged TH status during the early stages of embryonic development might also be the case in MCT8-deficient mammals. Recently, multiphasic changes of thyroid levels and function were found in the perinatal MCT8-KO mice. While hypothyroidism exists in the brain of adult MCT8-KO mice [13], [22], TH levels were similar to those of WT mice on embryonic day 17 (E17). Unexpectedly, hyperthyroidism appeared at ages E18 and P0, one day prior to birth and on the day of birth, respectively [22]. Thus, we propose that at early stages of development, such as 3 dpf in zebrafish and approximately 6–7 weeks in a human fetus, MCT8 might have a TH-independent cellular function that induces neurological deficiencies in zebrafish and possibly in humans. Nevertheless, since MCT8 is a TH transporter in mammals and zebrafish [3], [21], [76], the involvement of TH signaling in the regulation of the neurological deficiencies should be further tested during several stages of zebrafish development. In addition, TH alteration may be a tissue- and even cell-specific condition in 3 dpf *mct8*−/− embryos. Supporting this notion, we found that the expression of *thraa* and *thrab* is reduced in 3 dpf embryos suggesting reduced TH levels inside the cells. Furthermore, administration of T3 increased the expression of *p0* in WT-sibling but did not recover the expression of *p0* in *mct8*−/− embryos, suggesting that elimination of MCT8 altered TH levels within the glial cells. Altogether, MCT8 regulates myelin-related gene expression and circuit formation in early developed zebrafish embryos and, putatively, also in human embryos, by an unknown function and by facilitates cellular influx and efflux of TH (Fig. 9).

Clinical observations showed that delayed myelination is a prominent feature in AHDS [28]–[30], [77], [78]. Loss of myelin sheaths produces a wide variety of neurological symptoms, including the slow progression of action potential and deficient axon elaboration [79]. TH signaling is known to promote oligodendrocyte development and myelin production, thus, lower myelination is a key phenotype in the hypothyroid brain [80]–[83]. We therefore sought to examine the effect of MCT8 deficiency on the expression levels of *oligo2*, *p0*, and *mbp*, as well as on tissue distribution of glial cells in developing embryos. The zebrafish CNS is rich in oligodendrocytes, which express orthologs of mammalian genes involved in myelin formation, such as *olig2*, *p0*, and *mbp*. We found that the expression of these myelin-related genes was altered in *mct8*−/− embryos. These results suggest that loss of MCT8 enriched the number of neural precursor cells and delayed the development of mature oligodendrocytes and Schwann cells, and consequently, myelination. The mechanisms by which MCT8 regulates myelination could be explained by either direct effect on myelin-related genes, which results in impaired oligodendrocyte function, or indirect effect on axon maturation and processing that reduces the number of potentially myelinated axons [82], [84]. These MCT8-dependent myelin processes are likely partially regulated by TH signaling because TRs binding sites are located on the promoters of all three tested genes, and because TH induced the expression of *p0* in WT-sibling but not in *mct8*−/− embryos (Fig. 9).

Taking into account the deficiencies in behavioral performance and that deficient myelination affects axon processing [85], we examined the formation of specific neuronal circuits in *mct8*−/− larvae. In zebrafish, like in other vertebrates, axons branch dynamically throughout pathfinding; branches are added and eliminated, and successive branches typically project toward the target zone [86]. In previous work, we used MO knockdown strategy to show that the transient reduction of MCT8 alters the organization of neural cells in the brain and spinal cord. In severe cases of MO-injected embryos, the morphology of the larvae was abnormal [20]. Similarly, the *mct8*−/− larvae demonstrated altered neuronal development, but the general morphology was normal and the larvae were viable and fertile. This discrepancy is probably because of unspecific toxicity associated with the use of MO [87], which was undetectable in the ZFN-mediated stable mutant, further strengthening the use of the mutant methodological approach. Here, transgenesis and live-imaging enabled us to label specific sensory and motor neurons and measure different parameters of their arbor processes. Interestingly, we found that total arbor length was reduced because the number of mature branches was decreased. The reduction in the number of branches was linked to reduction in the number of filopodia. To understand the dynamics of filopodial plasticity, we performed time-lapse live imaging of single RB axon arbors. We found that the growth and branching of an axon arbor occurred by an iterative sequence of filopodial formation and elimination in both *mct8*−/− and WT-sibling embryos. Importantly, the rate of filopodial turnover in the *mct8*−/− was significantly reduced. Furthermore, filopodia number is increased under T3 administration. These results suggest that elimination of MCT8 stabilized filopodia dynamics, potentially through reduction in TH levels, which results in a reduced number of mature branches. Interestingly, the behavior and dynamics of axon growth in other zebrafish neuronal circuits, such as in the optic tectum, are reminiscent of the processes in RB axons [88], [89]. Thus MCT8 is likely involved in the mechanism that regulates axon arborization in the brain and spinal cord.

Ataxia and deficiencies in locomotor activity are prominent symptoms of AHDS patients [24], [90]. We therefore speculated that neuronal-circuit deficiencies occurred in the neuromuscular junction, particularly alterations in synaptic formation and plasticity. Using the presynaptic marker SYP-EGFP and live imaging, we quantified the synapse number. These experiments revealed reduction in synaptic density in the axons of the motor neurons. Synaptic morphology and number are closely linked to circuit function, and many psychiatric and neurological disorders, such as fragile X syndrome, are accompanied by alterations in synaptic connections [91], [92]. Hence, we suggest that reduced synaptic density in the motor neurons affects downstream behavioral performance of *mct8*−/− larvae, and potentially also human patients. The mechanism by which MCT8 regulates synaptic density may be mediated by TH, which controls the number and activity of synapses [93], [94]. These findings reveal a critical role of MCT8 in the regulation of synaptic density in motor neurons, and can have important implications for understanding behavioral abnormalities in AHDS patients.

The altered expression of myelin genes and deficient circuit formations could be the cause for the altered behavioral performance of *mct8*−/− larvae, and possibly also in humans. Indeed, AHDS patients exhibit severe hypotonia and develop a permanent severe mental and motor retardation, demonstrated by the inability to speak and walk independently [24], [90]. High-throughput video-tracking behavioral systems were used to show that the locomotor activity of *mct8*−/− larvae was reduced during both day and night. This reduction is partially because of their inability to reach maximum velocity. In addition, *mct8*−/− larvae demonstrate deficiency in their response to external light and dark stimuli. An intriguing explanation for this might involve both the motor and sensory neurons that regulate baseline locomotor activity and behavioral state transitions [95]. While reduced synaptic density in the motor neurons might inhibit baseline locomotor activity, altered formation of axon arbors in sensory neurons, such as RB neurons, might affect behavioral-state transitions. However, it is likely that other neurons, such as Mauthner cells [96], are also affected by MCT8 elimination and contribute to the behavioral deficiencies. Considering the diverse neurological and behavioral deficiencies exhibited by the *mct8*−/− larvae, we suggest that MCT8 mediates locomotor activity and the response to external stimuli via regulation of neuronal processing and synaptogenesis in specific circuits (Fig. 9).

The deficiencies in locomotor activity during both day and night raised the possibility that these larvae also exhibit sleep difficulties. The zebrafish is a diurnal vertebrate that exhibits neurological and behavioral characteristics of mammalian sleep and wakefulness [51], [55]–[58], [97]. Here, we found that loss of MCT8 increased sleep time and the number and length of sleep episodes during both day and night. This phenotype was robust and might also be present in other models for MCT8 deficiency. To date, there has been no report that characterized the sleep pattern in AHDS patients. However, this issue was occasionally raised by the families of patients (unpublished results) and should be monitored routinely across the lifespans of patients.

The options for therapeutic treatment for AHDS patients are limited. Application of treatment immediately after birth and even earlier, during pregnancy, is expected to best prevent neurological deficiencies. To date, treatments have attempted to normalize serum TH levels. It is not anticipated that TH treatment will help because in the absence of MCT8, TH transport into the brain is impeded. A promising approach is the use of TH analogs that can activate TH receptors but are not dependent on MCT8 for cellular entry [46], [47]. Indeed, DITPA has been tested in adult MCT8-KO mouse and administered to several AHDS patients [49], [50]. In both rodents and humans, the peripheral state of hyperthyroidism improved. However, DITPA treatment did not lead to a significant improvement of neurological parameters in patients. This can be explained by the relatively advanced age of the patients at the beginning of the treatment or an alternative TH-independent role for MCT8. Other therapeutic options for AHDS patients are the TH analogs TETRAC and TRIAC. Recently, assays on MCT8-KO and double MCT8/PAX8-KO mice demonstrated the potency of TETRAC in replacing TH during brain development [48]. These pilot studies are promising; however, it is still unclear to what extent the three analogs can replace TH during all stages of brain development and, importantly, direct comparison of the drugs in the same animal model that exhibits neurological deficiencies, was not performed. Here, we administered TH and TH analogs to *mct8*−/− embryos. Zebrafish provide an attractive system for high-throughput pharmacological screens because they can be easily treated with diverse drug concentrations at different time points, ranging from one-cell-stage to fully developed larvae. We found that at 3 dpf, all three TH analogs recovered the expression of *p0*, a key myelin-related gene, in *mct8*−/− embryos. In contrast, TH administration did not restore the expression of *p0*. These results suggest that myelin-related deficiencies can be treated using TH analogs. Further research is needed to pinpoint the most advanced stage in development that enables efficient treatment. In addition, these pharmacological studies should be expanded to include more drugs and targeted phenotypes. Indeed, we also found that TRIAC positively affect axon outgrowth in RB neurons. This type of experiments, together with the overexpression of candidate genes, such as *p0*, that might bypass specific deficient neurological pathways, will not only advance our understanding of the mechanism of the disorder but might also provide future pharmacological and gene-therapy approaches to treat psychomotor retardation.

Our study demonstrates the feasibility of monitoring myelin-related processes, structural synaptic plasticity, behavior, and the development of single axon arbors longitudinally in live MCT8-deficient animals, with application to the study of AHDS and other psychomotor retardation disorders. We found that the neurological deficiencies diverge and are circuit-specific. They are probably not unique to neurons in the spinal cord, and additional live-imaging experiments within the transparent zebrafish brain are required to elucidate specific altered brain circuits. The neurological alterations are associated with reduced locomotor activity, altered locomotor response, and increased sleep. The cellular mechanism that regulates these neurological and behavioral deficiencies is likely involved the transport of TH, but other functions of MCT8 cannot be ruled out, particularly at early stages of development. In future studies, there is a need to further evaluate the role of TH signaling in specific tissues and during late developmental stages in *mct8−/−* larvae. The recently developed genome editing approaches including ZFN, transcription activator-like effector nucleases (TALENs) and clustered regulatory interspaced short palindromic repeat (CRISPR)/Cas9, which became a straightforward technology in zebrafish [98] will enable productive study on the function of TH-transporters and AHDS in late developmental stages and in adults. In addition, an important future direction is to understand the genetic, neuroanatomical and behavioral similarities and variations between zebrafish and mammals. The recent development of MCT8/OATP1C1 double KO mice, which demonstrated neurological and behavioral abnormalities [99] provides the opportunity to study the mechanisms underlying AHDS in two vertebrate models. Translating these findings into comparatively large-scale pharmacological screens, gene therapy and stem-cell therapy will hopefully lead to the finding of a suitable treatment for AHDS and other psychomotor retardation disorders.

## Materials and Methods

### Zebrafish husbandry

Adult zebrafish were raised and maintained in fully automated zebrafish housing systems (Aquazone, Israel; temperature 28±0.5°C, pH 7.0, conductivity 300 µS) under 14 h light/10 h dark cycles, and fed twice a day. Embryos were produced by natural spawning and raised in egg-water containing methylene blue (0.3 ppm) in a light-controlled incubator at 28±0.5°C, as previously described [51]. All animal protocols were reviewed and approved by the Bar-Ilan University Bioethics Committee.

### Establishment of mct8 mutant line

Custom-designed ZFN plasmids and mRNA were commercially synthesized (Sigma-Aldrich, St. Louis, MO) to target a *HaeII* restriction site located in the first exon of the zebrafish *mct8* gene. Each ZFN array was designed to recognize 15 bp sequence upstream and downstream to the *HaeII* restriction site (5′-CGGCCACGCCGCCTGgcgctcACCCTGGACAAGGCT-3′, Fig. 1A). Approximately 100 ng/µl of each ZFN mRNA was co-injected into one-cell-stage WT embryos. These mosaic embryos were raised to adulthood and out-crossed with WT fish in order to identify F0 founder fish. F1 heterozygous fish, which carry a 7 bp deletion mutation in the targeted site, was selected and out-crossed with WT fish. The F2 heterozygous progeny were inter-crossed to generate the homozygous *mct8−/−* line (Fig. 1A, B).

Mutation screens and genotyping were conducted as follows: genomic DNA was extracted from 1 dpf embryos or a clipped tail fin of adult fish, using the KAPA express extract Kit (Kapa Biosystems Inc., Boston, MA) according to the manufacturer's instructions. Genomic DNA was then amplified by PCR using the following primers: 5′-gaggagttcgaggagcagga-3′ and 5′- caccagcatcatgtgcagaa-3′, and the 234 bp PCR product was digested with *HaeII* restriction enzyme. A digested PCR product was then run on 2% agarose gel. While complete digestion of WT DNA resulted in two short fragments of 104 bp and 130 bp, 234 bp PCR product was shown in *mct8−/−* fish, confirming the introduction of the mutation at the target site. When needed, this mutated DNA fragment was sequenced to confirm the presence of the 7 bp deletion. Heterozygous fish show three DNA fragments, indicating the presence of both mutated and WT *mct8* alleles (Fig. 1C).

### Cell culture and transient transfection

HEK293T Cell line were grown in Dulbecco's modified Eagle's medium (DMEM) containing 10% heat-inactivated fetal bovine serum (FBS) and 1% nonessential amino acids (Biological Industries, Beth Haemek, Israel), and incubated at 37°C and 5% CO2. HEK293T cells were transfected with 10 µg of either *pCS-cmv:MCT8-EGFP*, *pCS-cmv:MCT8mut-EGFP* or *pCS2-cmv:EGFP* DNA constructs using the calcium phosphate method. The culture medium was changed 6 h after transfection and cells were harvested 24 h later.

### Western blotting

Cells were lysed with RIPA buffer (20 mM Tris pH 7.5, 150 mM NaCl, 1 mM EDTA, 1% Nonidet P-40, 0.5% sodium deoxycholate, 2 mM Na3VO4, 1 mM NaF and 10 mM β-glycerophosphate) and supplemented with complete protease inhibitor cocktail (Roche Applied Science, Penzberg, Germany). Lysates were incubated on ice for 20 min and centrifuged at max speed for 10 minutes at 4°C and the supernatants were obtained. Protein concentration was measured by Bradford analysis. A total of 30 µg protein extract per lane were separated on 10% SDS polyacrylamide gel. After electrophoresis, proteins were transferred to nitrocellulose membrane (BIO-RAD, Hercules, CA, USA) and membrane was blocked for 1 h with 5% skim milk in PBST. Next, the blots were incubated for 1 h at room temperature with 5% skim milk in PBST containing anti-GFP primary antibody (GFP (B-2): sc-9996, Santa Cruz Biotechnology, Dallas, TX, USA), diluted 1∶1000. Following three washes, membranes were incubated at room temperature for 1 hr with 5% skim milk in PBST containing the secondary antibody (goat anti-mouse IgG-HRP: sc-2005, Santa Cruz Biotechnology, Dallas, TX, USA), diluted 1∶4000. Signals were visualized by SuperSignal West Pico Chemiluminescent Substrate according to the manufacturer instructions (Thermo Fisher Scientific, Waltham, MA, USA).

### Transgenic lines

Establishment of the *tg(uas:SYP-EGFP)* stable transgenic line was conducted using the Tol2 system [100]. In order to prepare the *pT2-uas:SYP-EGFP* construct, the upstream activation sequence (*uas*, kind gift of Prof. Philippe Mourrain, Stanford University) was double-digested with *StuI* and *EcoRI*, and ligated into a *StuI*/*EcoRI*-digested *pT2-hcrt:SYP-EGFP* vector [17], replacing the *hcrt* promoter.

To generate *tg(huc:GAL4Xuas:memYFP)* transgenic line on the background of *mct8* mutation, *tg(huc:GAL4Xuas:memYFP)* transgenic line (kindly provided by Dr. Bettina Schmid, Ludwig-Maximilians University Munich, Germany) was crossed with *mct8−/−* zebrafish. Next, Transgenic *tg(huc:GAL4Xuas:memYFP)/mct8+/−* and *mct8−/−* zebrafish were crossed to produce the *tg(huc:GAL4Xuas:memYFP)/mct8−/−* and *tg(huc:GAL4Xuas:memYFP)/mct8+/−* lines.

To generate the *tg(mct8:GAL4)*/*tg(uas:SYP-EGFP)* double transgenic line on the background of the *mct8* mutation, *tg(mct8:GAL4)* [20] and *tg(uas:SYP-EGFP)* were independently out-crossed with *mct8−/−* zebrafish. The resulting *tg(mct8:GAL4)*/*mct8+/−* and *tg(uas:SYP-EGFP)*/*mct8+/−* lines were crossed to produce the *tg(mct8:GAL4)*/*tg(uas:SYP-EGFP)*/*mct8−/−* and *tg(mct8:GAL4)*/*tg(uas:SYP-EGFP)*/*mct8+/−* fish lines.

### DNA constructs and transient expression assays

Transient expression assays of the following DNA constructs, *pT2-huc:Gal4-VP16* (kind gift of Prof. Thomas Misgeld, Technical University Munich, Germany), *pT2-uas:tRFP* (kind gift of Dr. Gordon Wang, Stanford University), *uas:memYFP* (kindly provided by Prof. Thomas Misgeld, Technical University Munich, Germany) and *pT2-uas:SYP-EGFP* were performed by microinjection of approximately 2 nl into one-cell-stage zebrafish zygotes, at a concentration of 20 ng/µl each, using micromanipulator and PV830 Pneumatic Pico Pump (World Precision Instruments, Sarasota, FL).

To prepare probes for whole mount ISH experiments, DNA fragments containing the coding region of the following genes: *klf9* (NM_001128729.1), *nrgna* (ENSDART00000057910), *mbp* (AY860977.1), *olig2* (NM_178100.1) and *p0* (NM_194361.2) were PCR-amplified using the following primers: *klf9*: 5′- atgacggacgtagatattgcagc-3′ and 5′-ttaaacaccagcagacatatg-3′; *nrgna*: 5′-atggactgtcgaaacgaagg-3′ and 5′-ctacttcggctcgcgttg-3′; *mbp*: 5′-atggccactgcaagcacctc-3′ and 5′tcagaagatggtgctccagcg-3′; *p0*: 5′-atgctgtccgtactggcact-3′ and 5′-tcagatacgctgtttttgctg-3′; *olig2*: 5′-atggactctgacacgagccgagtgt-3′ and 5′-tttgagtcactggtcagccgt-3′. All PCR products were cloned into a *pCRII-TOPO* vector (Invitrogen, Carlsbad, CA), linearized by *NotI* and served as a template to transcribe digoxigenin-labeled anti-sense RNA probes.

To prepare fusion constructs, WT and mutated *mct8* coding sequences were amplified by PCR using the following primers, 5′-taaccggaattccgccaccatgcactcggaaagcgat-3′ and 5′-taaccgaccggttatgtgtgtctccatgtccg-3′, and subcloned into *pCS2-cmv:EGFP* vector [101] using *EcoRI* and *AgeI*. The resulting *pCS2-cmv:MCT8-EGFP* and *pCS2-cmv:MCT8mut-EGFP* fusion constructs and the *pCS2-cmv:EGFP* vector were linearized with *XbaI* restriction enzyme, and *in vitro* transcribed using the mMESSAGE mMACHINE SP6 kit (Ambion Inc., Austin, TX). Approximately 2 nl of 100 ng/µl mRNAs were microinjected into fertilized one-cell-stage embryo.

To prepare *mct8* mRNA *in vitro*, the *mct8* full coding sequence was PCR-amplified using a platinum taq DNA polymerase (Life Technologies, Grand Island, NY) and the following primers: 5′-cgcggatccatgcactcggaaagcgatgacaac-3′ and 5′-cgcactagttcatatgtgtgtctccatgtccgtg-3′. The PCR product was subcloned into *pCS-TP* vector [100] using *BamHI* and *SpeI* restriction enzymes. Following linearization with *NotI*, *mct8* mRNA was *in vitro* transcribed using the mMESSAGE mMACHINE SP6 kit (Ambion Inc., Austin, TX), according to the manufacturer's instructions. Rescue experiments were conducted by the injection of approximately 2 nl volume of *in vitro* transcribed *mct8* mRNA (100 ng/µl) to one-cell stage *mct8−/−* embryos.

### Whole-mount ISH and immunohistochemistry assays

In both whole mount ISH and immunohistochemistry experiments, embryos and larvae were fixed in 4% paraformaldehyde overnight at 4°C, washed in PBST, and stored in 100% methanol. The location and level of mRNA expression were detected by whole-mount ISH, as described [51], [68]. Digoxigenin-labeled anti-sense RNA probes for *klf9*, *nrgna*, *mbp*, *olig2* and *p0* were generated from the vector templates described above using DIG RNA labeling kit (Roche, Indianapolis, IN), according to the manufacturer's instructions. Digoxigenin-labeled anti-sense RNA probes for *myoD* and *p2rx3.1* are the same as those described previously [20], [68]. All probes were used at a concentration of 0.5–1 ng/µl.

For double fluorescence ISH, fluorescein-labeled anti-sense RNA probe for *mct8* was transcribed from the vector template previously described [20], using Fluorescein RNA labeling kit (Roche, Indianapolis, IN). Digoxigenin-labeled *p2rx3.1* and fluorescein-labeled *mct8* anti-sense RNA probes (2 ng/µl) were simultaneously hybridized. Next, anti-fluorescein-POD antibody (Roche, Indianapolis, IN), diluted 1∶250, and anti-digoxigenin-AP antibody (Roche, Indianapolis, IN), diluted 1∶2500, were simultaneously incubated over-night at 4°C. *p2rx3.1* mRNA was visualized using TSA Plus Fluorescein System (Perkin-Elmer, Waltham, MA). *mct8* mRNA was subsequently visualized by an enzymatic reaction using Fast Red substrate (Roche, Indianapolis, IN).

Whole mount immunohistochemistry was carried out as previously described [20], using primary anti-MyHC for slow muscles antibody (F59, DSHB, USA; kind gift of Alon Daya and Prof. Stella Mitrani-Rosenbaum, Hebrew University of Jerusalem, Israel), diluted 1∶10, and a secondary goat anti-mouse Alexa Fluor 488 IgG (H+L) antibody (A-11029, Invitrogen, Carlsbad, CA), diluted 1∶250.

For double whole mount fluorescence immunohistochimestry-ISH labeling, ISH was conducted using Digoxigenin-labeled *p2rx3.1* and *p0* anti-sense RNA probes (2 ng/µl), following by detection using Fast Red substrate (Roche, Indianapolis, IN). Prior to counterstaining, larvae were washed 5 times in PBST, blocked with 20% goat serum diluted in PBST for 1 h at room temperature and incubated with rabbit anti-EGFP (SC-8334, Santa Cruz Biotechnology, Santa Cruz, CA) primer antibody, diluted 1∶250, in blocking buffer overnight at 4°C. Next, larvae were washed in PBST and blocked for 1 h. Anti-GFP antibodies were detected with a secondary goat anti-rabbit Alexa Fluor 488 IgG (H+L) antibody (A-11008, Invitrogen, Carlsbad, CA), diluted 1∶250.

### Real-time PCR quantification assays

Relative mRNA quantification of *klf9*, *nrgna*, *tsh*, *trh*, *dio1*, *dio2*, *dio3*, *mct10*, *oatp1c1*, *thraa*, *thrab*, *thrb*, *olig2*, *mbp and p0* was determined using qRT-PCR. Total RNA was extracted from 3 dpf embryos using the Direct-zol RNA MiniPrep kit (Zymo Research Corporation, Irvine, CA), according to the manufacturer's instructions. For each tested gene, a total of 5–15 biological samples were used. Each biological sample was contained a pool of 8–17 embryos. mRNA (1 µg) was reverse-transcribed using qScript cDNA SuperMix (Quanta BioSciences, Gaithersburg, MD). Relative transcript levels were determined by the 7900HT Fast Real-Time PCR System (Applied Biosystems, Foster City, CA). Triplicates of each cDNA sample were PCR-amplified using the PerfeCTa SYBR Green FastMix (Quanta BioSciences, Gaithersburg, MD) and the following specific primers: *klf9*: 5′-cgtttcatgtgcccactttg-3′ and 5′-tgtggatgttgaagagtgtcg-3′; *nrgna*: 5′-tggactgtcgaaacgaagg-3′ and 5′-accagcttgaatcttagcgg-3′; *tsh*: 5′-cccactgactacaccatctac-3′ and 5′-catcccctctgaacaataaaacg-3′; *trh*: 5′-gcagacccacagcatcag-3′ and 5′-caggccaagacgaacaca-3′; *dio1*: 5′-tgctttaattaccctggaccg-3′ and 5′-tgctgaagtccttgacaagc-3′; *dio2*: 5′-tggatgcctacaaacaggtg-3′ and 5′-ggcgatcaggagactcaaag-3′; *dio3*: 5′-ccagaagctggacttcttca-3′ and 5′-aagttgaggatcagcggtct-3′; *mct10*: 5′-tgtaacggctcggtgttc-3′ and 5′-aagatcatgcccatcgacag-3′; *oatp1c1*: 5′-tcatctcccaaggaaaacgag-3′ and 5′-agaaataggcgaaggacaagg-3′; *thraa*: 5′-ctgatgccatctttgatttggg-3′ and 5′-gtacatctcctgacacttctcg-3′; *thrab*: 5′-tctgatgccatcttcgacttg-3′ and 5′-gtacatctcctggcacttctc-3′; *thrb*: 5′-gctctggctcttatgacatgg-3′ and 5′-tcgctgatatctcgtgctttg-3′; *olig2*: 5′-cgagtgaactggaatagccttac-3′ and 5′-gctcgtgtcagagtccatg-3′; *mbp*: 5′-gaggagacaagaagagaaaggg -3′ and 5′-gaaatgcacgacagggttg-3′; *p0*: 5′-acctgtgatgccaagaacc-3′ and 5′-ttgccacaacgaggatca-3′; and *β-actin*: 5′-tgaatcccaaagccaacagag-3′ and 5′-ccagagtccatcacaataccag-3′. The relative quantification of each gene expression was normalized against *β*-actin mRNA expression levels and subjected to the ΔΔCT method [51].

### Pharmacological assays

In all pharmacological assays one-cell stage embryos were placed in glass Petri dishes (50–80 embryos per dish) containing either a specific drug or 5×10^−6^ M NaOH diluted in zebrafish water for control groups. The exposure medium (25 ml per dish) was exchanged twice a day. Stock solutions of 100 µM T3, T4, TETRAC, TRIAC (Sigma-Aldrich, St. Louis, MO) and DITPA (Santa Cruz Biotechnology, TX), were prepared in 0.05 M–0.1 M NaOH and diluted in zebrafish water to the final administered concentrations. In order to choose the appropriate working dilution for each substance, a preliminary dose-dependent assay was performed using WT embryos. Four to five different concentrations in the range of 0.5 nM to 100 nM were tested for each substance. In addition, a control group of embryos was raised in 5×10^−5^ M NaOH, the highest NaOH concentration applied to the treated groups. During the experiments, embryos were screened for morphological developmental abnormalities, such as distorted body shape, pigmentation defects, and movement disabilities. The highest substance concentration that lacked morphological defects (0.5 nM for T3, T4, TETRAC, TRIAC and 5 nM for DITPA) was chosen as the working dilution for all pharmacological experiments.

### Bioinformatical promoter analyses

Sequences of 7, 5, and 2 kb upstream to the 5′ UTR of *olig2* [102], *p0* [103], and *mbp* [104] genes, were analyzed using the University of California, Santa Cruz (UCSC) Genome Browser website. The prediction of TREs within the putative promoters was performed using the RXRA::VDR matrix of the Jaspar database tool (http://jaspar.genereg.net/). In general, TREs were identified as two or more hexamer (A/G)GGT(C/A)A consensus sequences arranged in tandem arrays [39], [40]. One putative site was predicted in the *p0* and *mbp* promoters, and three putative sites were predicted in the *oligo2* promoter; the one site that demonstrated the highest similarity to the consensus sequence was chosen.

### Behavioral assays

At 6 dpf, *mct8*−/− larvae and their WT siblings were placed, individually, in 48-well plates under 14 h light/10 h dark cycles. Larva-containing plates were placed in the Noldus DanioVision tracking system (Noldus Information Technology, Wageningen, Netherlands) and acclimated for one hour prior to activity recording. Light intensity in the tracking system was 70 LUX (25% in the operating software) for all experiments. To monitor rhythmic activity during a daily cycle, larvae were maintained under the same light-dark regime prior to the experiment. To monitor responses to light/dark transitions, larvae were subjected to 3 intervals of 30 min light/30 min darkness. For each experiment, live video-tracking and analysis were conducted for 3 independent assays, using the EthoVision XT 9 software (Noldus Information Technology, Wageningen, Netherlands), as previously described [51]. Data analyses of total activity, velocity and sleep were performed according to the threshold parameters described previously [51].

### Imaging

Whole mount ISH-stained larvae were placed in 100% glycerol, and imaged from dorsal or lateral view using an epifluorescence stereomicroscope (Leica M165FC). Pictures were taken using Leica Application Suite imaging software version 3.7 (Leica, Wetzlar, Germany). In live imaging experiments, embryos and larvae were anesthetized with Tricaine (0.01%) and placed in low-melting-point agarose (1.0–2.0%) in a specially designed chamber filled with egg-water. Similar mounting protocol was used to image fixed embryos subjected to fluorescence ISH or immunohistochemistry. Confocal imaging was performed using a Zeiss LSM710 upright confocal microscope (Zeiss, Oberkochen, Germany). All images were processed using ImageJ (National Institutes of Health, Bethesda, MD) and Adobe Photoshop (San Jose, CA) software.

Calculation of total arbor length, number of branches, average branching length, and the number of filopodian branches in axon arbors of single motor neuron and RB neurons was performed using NeuronJ plugin in ImageJ software (National Institutes of Health, Bethesda, MD). Synaptic density was calculated by quantification of synapse number per 50 µm in the axonal arbor of single motor neuron or RB neurons, using ImageJ software (National Institutes of Health, Bethesda, MD).

In the time-lapse experiments, embryos were placed in a specially designed chamber with constant egg-water flow at a temperature of 28±0.5°C. Automatic imaging of several embryos was performed simultaneously using the ZEN 2011 software (Zeiss, Oberkochen, Germany), and up to 40 optical sections of 2 µm each were obtained for each embryo every 15 min, during 2.5 h.

## Supporting Information

Figure S1 *mct8* is expressed in oligodendrocytes. **A**. Dorsal view of whole mount *in situ* hybridization (ISH) in 3 dpf larvae shows *p0* expression in the midline of the hindbrain and midbrain. **B–D**. Dorsal view of 3 dpf *mct8:EGFP* embryos. Whole-mount ISH and immunofluorescence revealed co-localization (marked with arrows) of *p0* (red) and EGFP (green).
(TIF)

Figure S2 Swimming velocity is decreased in *mct8−/−* larvae during day and night. **A–B**. Velocity recording was performed in 6 dpf *mct8−/−* and their WT siblings larvae throughout a daily cycle under a 14 h light/10 h dark cycle. **A**. Maximum velocity [the maximum swimming distance (cm) in one second per each minute] was monitored. White and black horizontal boxes represent day and night, respectively. **B**. The average total maximum velocity of each genotype was measured during day and night. Values are represented as means±SEM (standard error of the mean). Statistical significance was determined by *t*-test: two-sample assuming unequal variances (** *p<0.001*).
(TIF)

Figure S3 MCT8 elimination does not affect muscle structure. **A–H**. Lateral view of the trunk of 3 dpf (**A, B, E** and **F**) and 6 dpf (**C, D, G**, and **H**) *mct8−/−* and their WT-siblings larvae. **A–D**. Immunostaining with an antibody against slow muscle myosin (F59). **E–H**. Whole-mount ISH using an mRNA probe against the muscle-specific marker *myod*.
(TIF)

Figure S4 The TH T3 increases the number of filopodian branches. The number of filopodian branches was quantified in the axons of the RB neurons (as shown in Fig. 8). At the one-cell-stage, WT embryos were treated with 0.5 nM T3. At 2 dpf, treated and untreated embryos were imaged. Values are represented as means ±SEM (standard error of the mean). Statistical significance was determined by *t*-test: two-sample assuming unequal variances followed by one-sample Kolmogorov-Smirnov test to assume normal distribution (*p<0.05).
(TIF)

Video S1 Filopodia dynamics in RB neurons. At 2 dpf, live imaging of filopodia turnover was performed in embryos co-injected with *huc:GAL4* and *uas:tRFP* constructs during 135 min. Green and red arrows mark new and lost filopodia, respectively.
(AVI)
