# Supplementary figures and images for: Altered Behavioral Performance and Live Imaging of Circuit-Specific Neural Deficiencies in a Zebrafish Model for Psychomotor Retardation
Source: PLoS Genet. 2014 Sep 25;10(9):e1004615. doi: 10.1371/journal.pgen.1004615 (PMC4177677; doi:10.1371/journal.pgen.1004615)

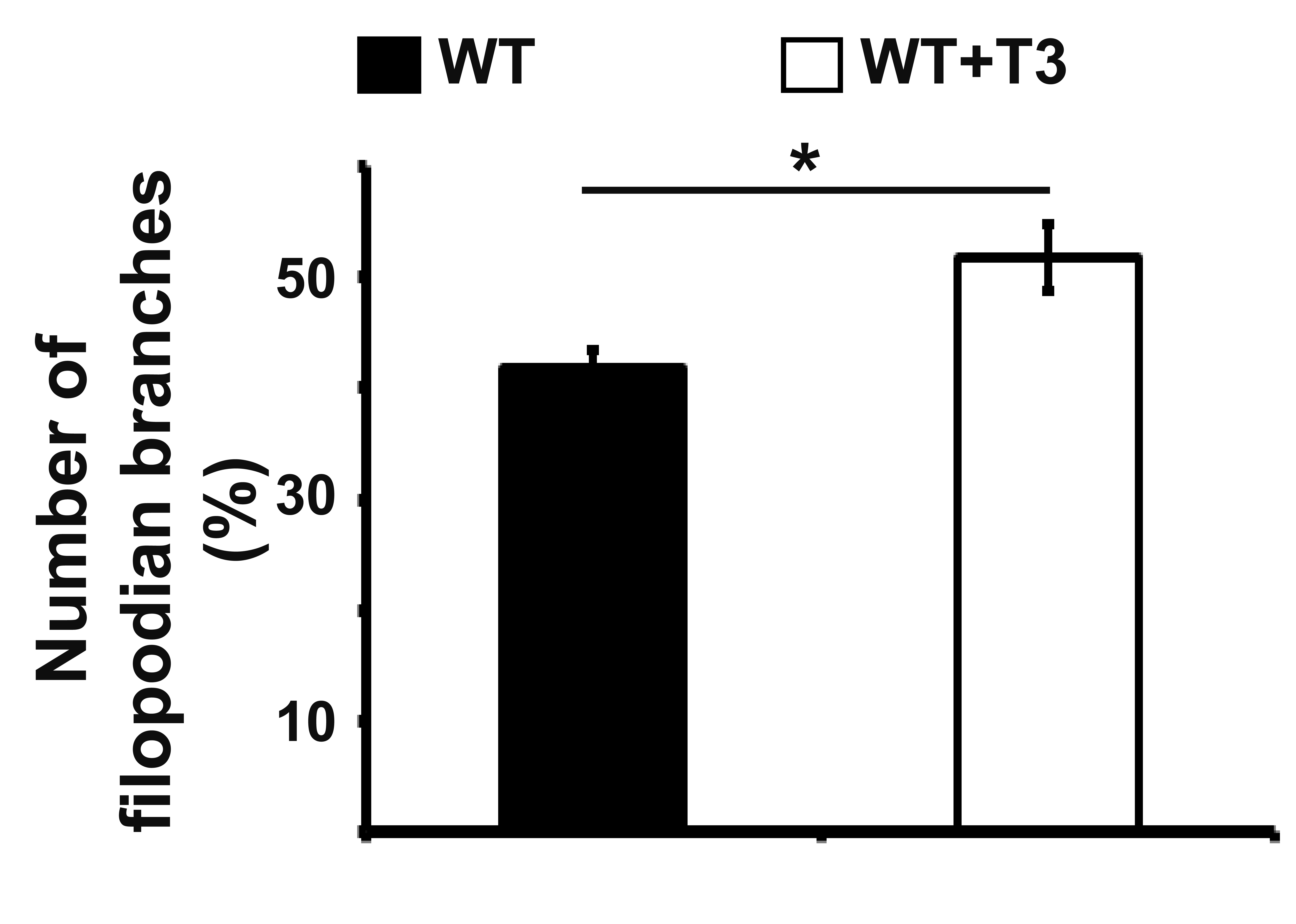

Supplement: Figure S4 — The TH T3 increases the number of filopodian branches. The number of filopodian branches was quantified in the axons of the RB neurons (as shown in Fig. 8). At the one-cell-stage, WT embryos were treated with 0.5 nM T3. At 2 dpf, treated and untreated embryos were imaged. Values are represented as means ±SEM (standard error of the mean). Statistical significance was determined by t-test: two-sample assuming unequal variances followed by one-sample Kolmogorov-Smirnov test to assume normal distribution (*p<0.05). (TIF) [file pgen.1004615.s004.tif]
